# MgP_4_/CNT‐Graphene Embedded in Hard Carbon Matrix as a High‐Capacity Anode for Next‐Generation Sodium‐Ion Batteries

**DOI:** 10.1002/advs.76701

**Published:** 2026-07-17

**Authors:** Sion Ha, Doyeon Lee, Dong Won Kim, Won‐Sik Kim, Minkyu Lee, Seong‐Hyeon Hong, Kyeong‐Ho Kim

**Affiliations:** ^1^ Department of Materials Science and Engineering Pukyong National University Busan Republic of Korea; ^2^ Department of Materials Science and Engineering and Research Institute of Advanced Materials Seoul National University Seoul Republic of Korea; ^3^ Department of Eco‐Friendly Energy Technology Pukyong National University Busan Republic of Korea; ^4^ Department of Physical Medicine and Rehabilitation Northwestern University Chicago Illinois USA

**Keywords:** anode material, hard carbon, high capacity, magnesium phosphide (MgP_4_), sodium ion battery

## Abstract

Sodium‐ion batteries (SIBs) are promising low‐cost alternatives to lithium‐ion batteries (LIBs), but their energy density remains limited. Hard carbon (HC) offers only modest capacity, while high‐capacity LIB anode materials, notably silicon (Si), are ineffective in SIBs due to the unfavorable thermodynamics of Na‐rich alloy formation. Here, we report a scalable mechanochemical synthesis of magnesium tetraphosphide (MgP_4_) and demonstrate that hybrid carbon matrix engineering enables durable and high‐capacity anodes. A two‐step multi‐walled carbon nanotube (MWCNT, T)/graphene(G) assembly, denoted as T2G1 (2:1 T:G by weight), where CNTs are introduced prior to graphene, constructs a continuous conductive and mechanically robust network. In contrast, reversing the assembly order (G2T1) leads to fragmented conductive pathways and inferior structural stability. Multiscale analyses, including cross‐sectional resistance mapping and structural characterization, reveal that the optimized matrix promotes uniform charge transport and suppresses structural degradation during cycling. As a result, the MgP_4_/T2G1 anode delivers stable high‐rate performance, retaining 468.5 mAh g^−1^ over 500 cycles at 1000 mA g^−1^ (85.3% retention). Furthermore, integrating 30 wt.% MgP_4_/T2G1 into commercial HC yields a practical composite with a reversible capacity of 146.5 mAh g^−1^ after 2000 cycles at 1000 mA g^−1^, corresponding to ∼2.6 times higher capacity than that of pristine HC electrode.

## Graphical abstract

Phosphorus‐rich MgP_4_ is introduced as a high capacity anode for sodium‐ion batteries (SIBs) via hybrid carbon matrix engineering. A sequential CNT/G assembly forms a conductive and mechanically robust network, delivering a high reversible capacity of 1386 mAh g^−1^ while stabilizing alloying reactions. Furthermore, integration with hard carbon enables improved cycling stability and practical applicability.

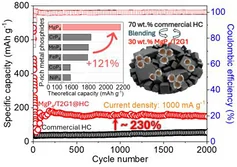

## 1 Introduction

The accelerating global demand for low‐cost, scalable, and sustainable energy storage has propelled sodium‐ion batteries (SIBs) into the spotlight as a viable alternative to lithium‐ion batteries (LIBs) [1, 2]. With the rapid market expansion of low‐cost LiFePO_4_ (LFP)‐based LIBs, there is growing interest in even more economical systems where sodium, rather than lithium, serves as the charge carrier. Owing to the natural abundance and wide geographical distribution of sodium resources, SIBs offer significant cost advantages and enhanced resource security, making them particularly attractive for grid‐scale energy storage systems (ESSs) and entry‐level electric vehicles (EVs) [3−5].

However, the practical implementation of SIBs is hindered by the intrinsic physicochemical properties of Na^+^ ions. Compared to Li^+^ ions, Na^+^ ions possess a larger ionic radius (1.02 vs. 0.76 Å) and a higher standard redox potential (−2.71 vs. −3.04 V vs. SHE), which lead to severe volume changes, sluggish ion diffusion kinetics, and lower energy density during cycling [6, 7]. These limitations not only degrade electrochemical performance but also necessitate the development of optimized electrode materials. Unfortunately, many conventional electrode materials used in LIBs, particularly anode materials, exhibit poor electrochemical performance when directly applied to SIB systems, posing significant challenges to their practical deployment.

Among various anode candidates, graphite, the most successful anode material in LIBs, exhibits negligible practical Na^+^ ion storage capability due to its unfavorable thermodynamics and structural incompatibility [8, 9]. The larger ionic radius of Na^+^ ion and its weaker interaction with graphene layers hinder the formation of stable Na–graphite intercalation compounds (GICs), unlike LiC_6_ in LIBs. As a result, only solvated Na^+^ ion co‐intercalation occurs in ether‐based electrolytes, underscoring the intrinsic incompatibility of graphite with Na^+^ ions [10, 11]. However, this mechanism relies on ether‐based solvents with poor oxidative stability, limiting compatibility with high‐voltage cathodes and reducing practical applicability. Consequently, hard carbon (HC) has emerged as the most promising alternative due to its enlarged interlayer spacing and disordered structure, enabling Na^+^ ions storage through adsorption and intercalation [12, 13]. Nevertheless, its low initial Coulombic efficiency (ICE), moderate capacity (∼300 mAh g^−1^), and limited rate capability of HC hinder its ability to meet the energy density requirements of next‐generation SIBs [14, 15].

This limitation parallels the early development of LIBs, where the capacity constraint of graphite was overcome by incorporating high‐capacity alloying materials such as silicon (Si), and silicon sub‐oxides (SiO_x_) into composite anodes [16−18]. Inspired by this strategy, integrating hard carbon with high‐capacity components has emerged as an effective approach to simultaneously enhance energy density, cyclability, and practical applicability in SIB systems. Among high‐capacity anode candidates for SIBs, Si, a benchmark material in LIBs, has attracted attention owing to its abundance and high theoretical capacity (∼3590 mAh g^−1^ for Li_15_Si_4_) [18, 19]. However, in contrast to LIBs, Si reacts with Na^+^ ions to form only NaSi, resulting in a much lower theoretical capacity (∼954 mAh g^−1^) and poor reversible performance due to sluggish sodiation kinetics and limited phase reversibility [20].

Alternatively, red phosphorus (P) has emerged as a promising alloy‐type anode owing to its high theoretical capacity (≈2596 mAh g^−1^ for Na_3_P) and low operating potential [21]. Nevertheless, its extremely low electronic conductivity (∼10^−14^ S cm^−1^), large volume expansion (∼300%), and unstable solid–electrolyte interphase (SEI) lead to rapid capacity fading and safety concerns, limiting its practical applicability [22, 23]. To overcome these limitations, metal phosphides (MP_x_) have been widely investigated, as they can buffer volume changes and enhance electrical conductivity by forming metallic phases during electrochemical sodiation [24]. In these systems, metal cations are electrochemically reduced to metallic states (M^0^) while P reacts reversibly with Na^+^ ions to form Na_3_P phase, leading to enhanced electrochemical reversibility and cycling stability compared to red P alone [25−27].

Among various MP_x_ systems, P‐rich phosphides (MP_x_, x ≥ 2) exhibit higher theoretical capacities due to their increased P contents in the compositions. Transition‐metal phosphides (TMPs) such as NiP_3_, CoP_3_, FeP_4_, MnP_4_, and CrP_4_ have demonstrated promising high capacities (1400–1829 mAh g^−1^), moderate electrical conductivity, and good cost‐to‐performance balance [28−32]. In general, the theoretical capacity of TMPs increases as the molar weight of the used TM decreases, but the differences among TMs (Cr, Mn, Fe, and Co) are minor due to their similar molar weights. Extending this concept to lighter elements provides a pathway to enhance theoretical capacity further. In this regard, magnesium tetraphosphide (MgP_4_), composed of lightweight and earth‐abundant Mg, is a particularly attractive candidate with a theoretical capacity exceeding 2000 mAh g^−1^.

However, such P‐rich high‐capacity anodes undergo severe volume changes during cycling, leading to structural instability, particle pulverization, and delamination [33, 34]. Therefore, a stable carbon matrix is essential to buffer these stresses and maintain electronic pathways for reversible cycling processes. Various carbon materials, including carbon black (CB), carbon nanotubes (CNTs), and graphene nanosheets (GNs), have been employed to construct conductive matrices for alloy‐type anodes. CB is widely used to enhance electrical contact but provides limited mechanical reinforcement, whereas CNTs offer one‐dimensional (1D) conductive pathways with improved flexibility, and GNs can form two‐dimensional (2D) conductive networks with high mechanical strength [35−37]. Despite these advantages, most studies rely on a single type of carbon component, which often results in insufficient structural stability and incomplete electron transport networks. In contrast, integrating multiple carbon structures, particularly hybrid networks combining 1D CNTs and 2D graphene, can potentially provide synergistic improvements in conductivity and mechanical robustness [38]. However, such interdimensional carbon matrices have rarely been explored for stabilizing P‐rich, high‐capacity anodes for SIBs.

In this study, we present a high‐capacity MgP_4_ anode that exploits the earth abundance, low density, and high chemical reactivity of Mg, offering facile and rapid synthesis while delivering a high theoretical capacity of 2170 mAh g^−1^ for SIBs. Unlike conventional solid‐vapor synthesis routes that require high temperature, high pressure, and prolonged reaction times [38, 39], MgP_4_ was synthesized via a high‐energy mechanochemical milling (HEMM) process, enabling rapid and scalable solid‐state synthesis under ambient conditions. To address the intrinsic instability of P‐rich anodes, we further developed a two‐step carbon‐engineering strategy in which MgP_4_ nanoparticles are encapsulated within a hybrid CNT/graphene network. These interdimensional structures effectively bridge neighboring particles, enhancing both electrical conductivity and mechanical integrity. As a result, the MgP_4_/CNT/G (MgP_4_/TG) composite demonstrates improved electrochemical performance and structural stability, highlighting its potential as a next‐generation anode material for SIBs. Furthermore, by integrating 30 wt.% MgP_4_/TG with commercial HC, we fabricated a MgP_4_/TG@HC composite electrode analogous to graphite/silicon composite anodes in commercial LIBs, achieving enhanced energy density and practical applicability in SIB systems.

## 2 Results and Discussion

### 2.1 Phase and Morphology of MgP_4_

A facile HEMM process was employed to synthesize the MgP_4_ phase and subsequently fabricate MgP_4_/TG@HC composites as illustrated in Scheme 1. Stoichiometric mixtures of micron‐sized Mg and red P powders (Mg:p = 1.0:4.0) were loaded into a milling vial and subjected to ball milling with a rotation speed of 400 rpm. Before milling, the x‐ray diffraction (XRD) pattern of the hand‐mixed powders shows diffraction peaks corresponding to the hexagonal structure of Mg, while red P remains amorphous (Figure S1). After 2 h of milling, the diffraction peaks can be indexed to the monoclinic MgP_4_ phase with a space group of P2_1_/c (PDF # 04‐001‐3774), indicating successful phase formation. Prolonged milling beyond this time leads to partial decomposition and formation of an impurity phase.

**SCHEME 1:**
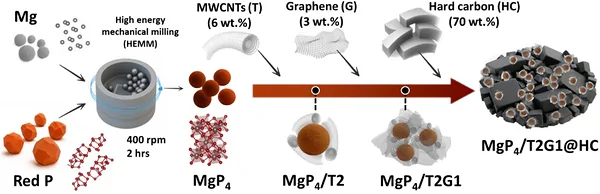
Synthesis process of MgP_4_/T2G1@HC composites.

Figure 1a shows the synchrotron x‐ray diffraction (SXRD) pattern of as‐synthesized MgP_4_ phase obtained after 2 h of milling, along with Le Bail fitting refinement for structural analysis. The refined lattice parameters are in good agreement with the ICDD reference (PDF # 04‐001‐3774), confirming the successful formation of phase‐pure MgP_4_ with reliable fitting results (Table S1). The crystal structure of MgP_4_ is illustrated in Figure 1b. In this monoclinic structure, Mg atoms are coordinated with six P atoms to form edge‐sharing MgP_6_ octahedra, which are arranged in a zigzag configuration along the crystallographic *c*‐axis.

**FIGURE 1:**
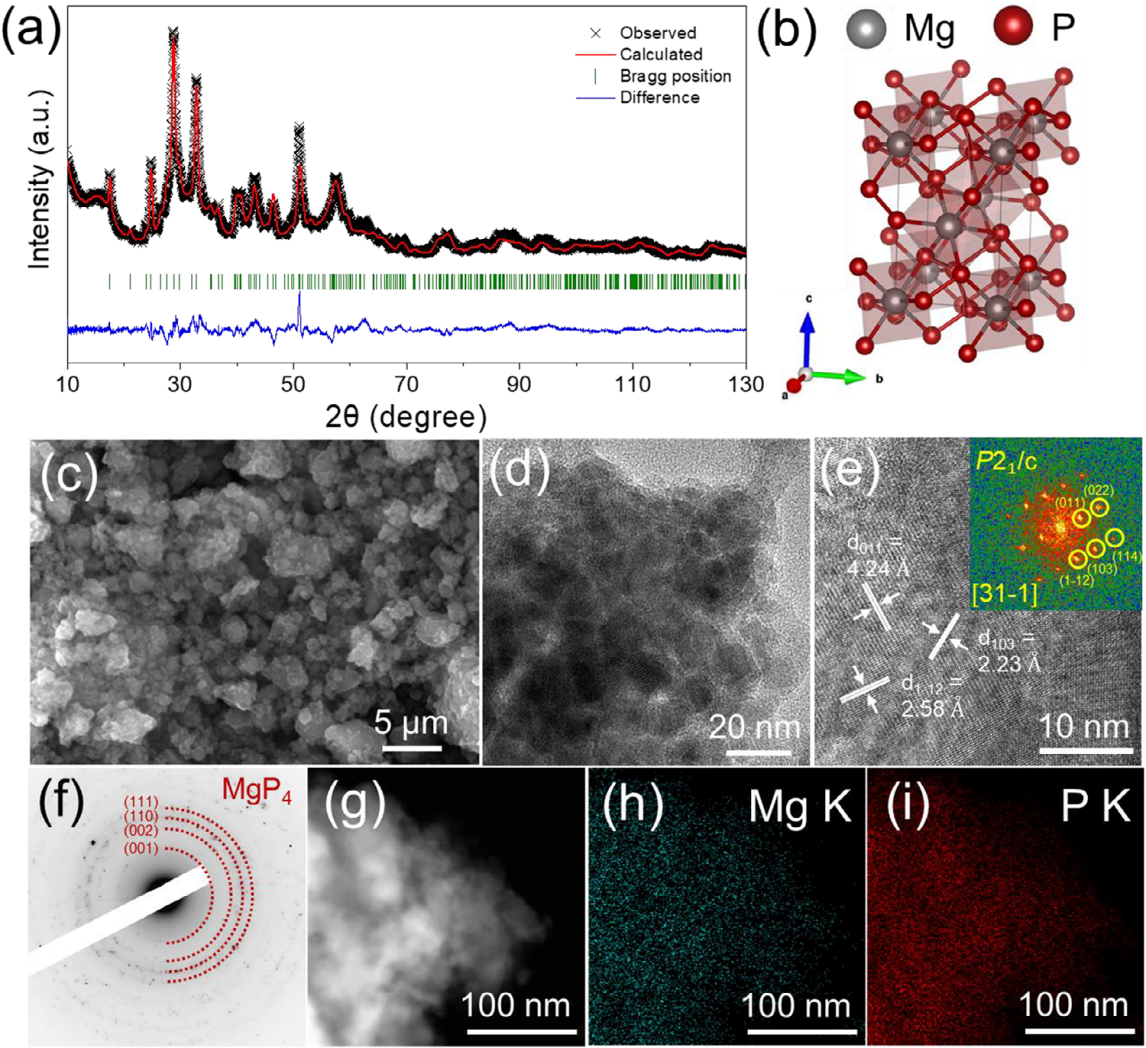
(a) Synchrotron XRD pattern and refined results, (b) crystal structure, (c) SEM image, (d) TEM image, (e) HRTEM image with FFT pattern, (f) SAED pattern, (g) HAADF‐STEM image and EDS mapping images of (h) Mg K and (i) P K for as‐synthesized MgP_4_ particles, respectively.

The morphology of as‐synthesized MgP_4_ powders was characterized by scanning electron microscope (SEM), revealing highly agglomerated secondary particles with sizes ranging from submicron to several micrometers (Figure 1c). The low magnification transmission electron microscope (TEM) image (Figure 1d) shows that these aggregates consist of densely packed nanocrystallites with sizes of approximately 10–20 nm. High‐resolution (HR) TEM image (Figure 1e) displays clear lattice fringes corresponding to the (011), (1‐12), and (103) planes of monoclinic MgP_4_, consistent with the fast Fourier transform (FFT) pattern along the [31‐1] zone axis. The selected area electron diffraction (SAED) pattern (Figure 1f) exhibits distinct diffraction rings indexed to the (001), (002), (110), and (111) planes of MgP_4_, confirming the polycrystalline nature of the sample. Furthermore, high‐angle annular dark field (HAADF) scanning transmission electron microscope (STEM) image (Figure 1g) and corresponding energy dispersive spectroscopy (EDS) elemental maps of Mg K and P K (Figure 1h,i) demonstrate the uniform distribution of Mg and P throughout the nanoparticles, indicating their compositional homogeneity.

### 2.2 Electrochemical Reaction Mechanism and Performance of MgP_4_

To evaluate the electrochemical sodium storage behavior of MgP_4_ electrode, galvanostatic discharge–charge tests were performed using a MgP_4_/Na^0^ half‐cell configuration (Figure 2a,b). At a current density of 50 mA g^−1^ within a voltage window of 0.01–3.0 V (vs. Na/Na^+^), MgP_4_ electrode delivered a high initial sodiation capacity of 1882.1 mAh g^−1^. However, due to the low initial coulombic efficiency (ICE) of 57.0%, the corresponding first desodiation capacity was limited to 1072.6 mAh g^−1^ (Figure 2a). The differential capacity plots (dQ dV^−1^) revealed similar cathodic peaks at 0.19 and 0.11 V (vs. Na/Na^+^) during the first and subsequent sodiation processes, while anodic peaks were observed at 0.46 and 0.63 V (vs. Na/Na^+^) during the desodiation processes (Figure 2b).

**FIGURE 2:**
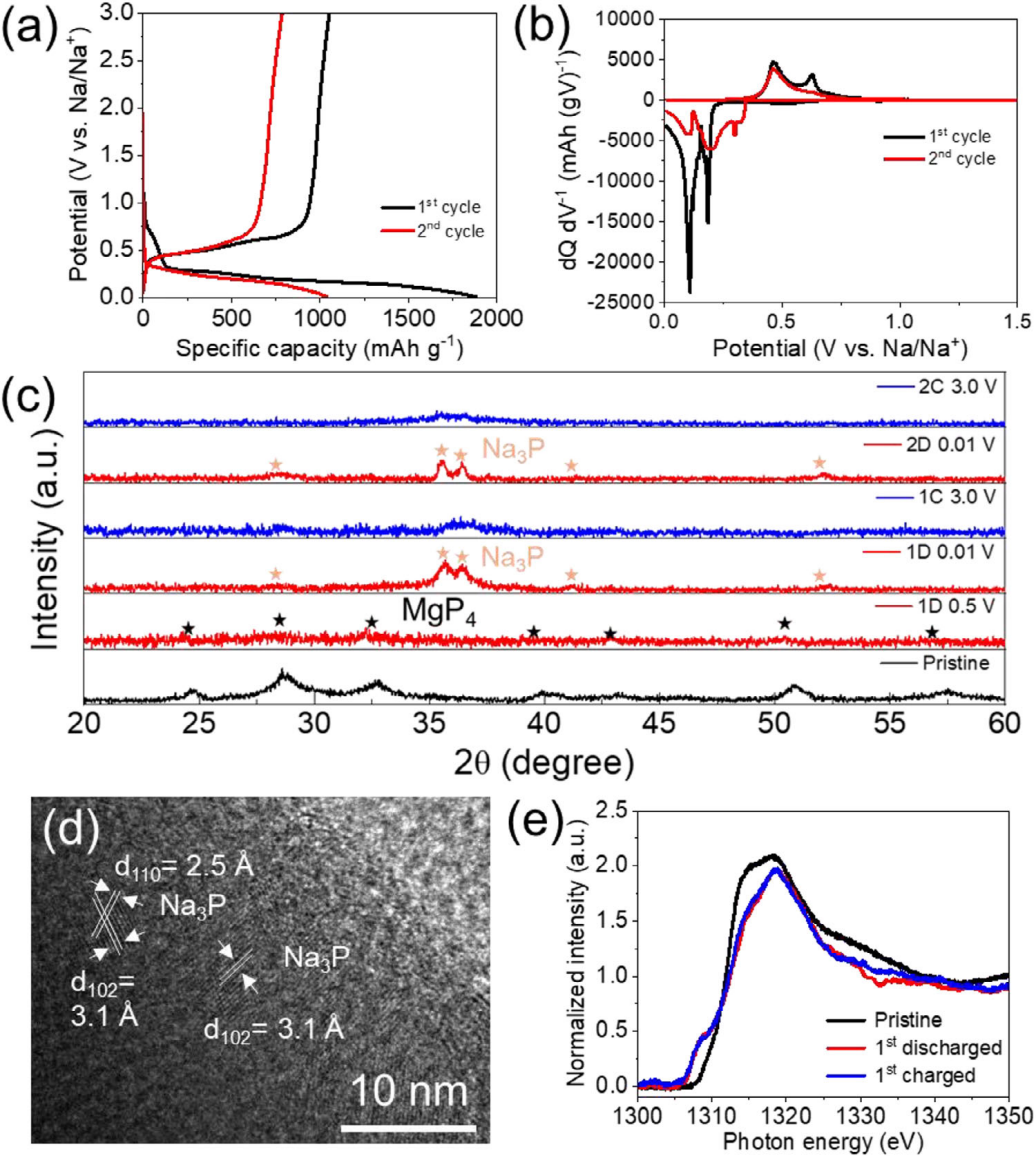
(a) Galvanostatic discharge/charge voltage profiles, (b) corresponding differential capacity plots (DCPs), and (c) ex situ XRD patterns at different discharge and charge states of MgP_4_ electrodes for SIBs. (d) HRTEM image for the first discharged state and (e) Mg K‐edge XANES spectra of MgP_4_ electrodes for pristine, first discharged and charged states. The reference XRD peaks corresponding to MgP_4_ (PDF #04‐003‐4161) and Na_3_P (PDF #01‐073‐3917) phases are marked with black and light red stars, respectively. The used electrolyte was 1.0 M NaClO_4_ in EC/DMC (1:1, v/v) with 5 vol.% FEC.

To elucidate the electrochemical reaction mechanism of MgP_4_ electrode, *ex‐situ* XRD measurements were conducted at different states of discharge and charge (Figure 2c). Upon discharge to 0.5 V vs. Na/Na^+^, the characteristic peaks of MgP_4_ significantly diminished without the appearance of new crystalline phases. Further discharge to 0.01 V resulted in the appearance of Na_3_P (PDF # 01‐073‐3917) phase, indicating the formation of sodiated products. This observation was further supported by *ex‐situ* TEM analysis (Figure 2d), where clear lattice fringes corresponding to Na_3_P were observed. To further investigate the reaction products, Mg K‐edge x‐ray absorption near‐edge structure (XANES) spectra was obtained (Figure 2e). Upon full discharge to 0.01 V, the appearance of a pre‐edge feature at 1307 eV confirmed the formation of metallic Mg^0^ via a conversion reaction (Figure 2e). Notably, the XANES spectra showed negligible changes upon charging to 3.0 V, indicating that Mg^0^ was not reconverted into original MgP_4_ phase but instead remained as a separate phase in Mg^0^/P composites. Correspondingly, the XRD pattern after charging remained largely amorphous, suggesting that Mg and P existed as nanoscale domains rather than reforming the crystalline MgP_4_ structure. During the subsequent sodiation, the Na_3_P phase reappeared, while it also remained in an amorphous state after the following desodiation process. Collectively, these results indicated that the overall electrochemical sodiation/desodiation of MgP_4_ electrode could be described as:

First sodiation: MgP_4_  +  12 Na^+^   +  12 e^−^  →  Mg^0^  +  4 Na_3_P

Subsequent desodiation/sodiation: Mg^0^  +  4 Na_3_P ↔ Mg^0^  +  12 Na^+^   +  12 e^−^  +  4 P^0^

The electrochemical Li^+^ ion storage behavior of MgP_4_ electrode was further examined using a MgP_4_/Li^0^ half‐cell configuration. Compared to the sodium system, the lithium cell exhibited lower initial discharge and charge capacities of 810 and 341 mAh g^−1^, respectively (Figure S2a,b). Although the overall reaction followed a similar conversion reaction mechanism, *ex‐situ* XRD and TEM analyses revealed that the reduced Mg° further alloyed with Li^+^ ion to form Li_0.8_Mg_0.2_ (PDF # 00‐152‐3818), while P was converted into Li_3_P phase (PDF # 00‐221‐2780), respectively (Figure S2c,d).

### 2.3 Improvement of Electrochemical Performance of MgP_4_ by Applying Hybrid CNT–G Matrix

To improve the low ICE and limited reversible capacity of MgP_4_, the incorporation of conductive carbon matrices was explored by enhancing both structural stability and electrical conductivity. To mitigate the severe volume changes during sodiation/desodiation and limited charge transport in pristine MgP_4_, various carbonaceous materials with different dimensionalities were employed to fabricate the composite electrodes. For initial screening, CB, MWCNTs, and GNs were individually incorporated at a fixed loading of 20 wt.% relative to MgP_4_. The resulting composites were denoted as MgP_4_/C2, MgP_4_/T2, and MgP_4_/G2, where C, T, and G represented CB, MWCNTs, and GNs, respectively, and the number indicated the carbon content in tens of wt.%. The composites were evaluated at a current density of 50 mA g^−1^. Under these conditions, the MgP_4_/T2 composite exhibited the highest reversible capacity among the tested samples, although its cycling stability remained insufficient for practical application (Figure S3a). Increasing the CNT content to 30 wt.% improved cycling stability but led to a noticeable decrease in reversible capacity of MgP_4_/T3 (Figure S3b). To balance capacity and cycling stability, hybrid carbon matrices were further invesitaged by fixing the total carbon content at 30 wt.% while varying the CNTs/GNs ratio. Notably, the composite prepared by sequential milling, initially incorporating 20 wt.% CNT followed by 10 wt.% GN (MgP_4_/T2G1), delivered a higher reversible capacity than the 30 wt.% CNT counterparts (MgP_4_/T3). In contrast, the inverse configuration (MgP_4_/G2T1) showed negligible improvement compared to MgP_4_/T3 (Figure S3b). To elucidate the origin of the improved electrochemical performance in this optimized carbon structure, further structural, chemical, and morphological analysis were conducted.

In the XRD pattern of as‐prepared MgP_4_/T2G1 particles, the characteristic peaks of monoclinic MgP_4_ (PDF #04‐001‐3774) were clearly observed, along with a broad peak around 26°, corresponding to the characteristic peaks of MWCNTs and GNs (Figure 3a,b). This indicated that no impurity phases were formed during the HEMM process used for composite fabrication. The elemental distributions of Mg and P within the T2G1 carbon matrix were further investigated by EDS. The results revealed a homogeneous spatial distribution of Mg, P, and C throughout the composite (Figure 3c–f), suggesting effective mixing between MgP_4_ and carbonaceous components. To further elucidate the structural configuration and local arrangement of carbon phase, additional Raman spectroscopy, SEM, and TEM analyses were performed. The Raman spectra of commercial MWCNTs and GNs exhibited characteristic D (∼1350 cm^−1^), G (∼1580 cm^−1^), and 2D (∼2700 cm^−1^) bands, which are associated with structural disorder, graphitic sp^2^ bonding, and a double‐resonant two‐phonon mode, respectively (Figure 3g) [41]. The I_D_/I_G_ ratio reflects the degree of disorder and graphitization, while the I_2D_/I_G_ ratio qualitatively indicates interlayer coupling and stacking [42, 43]. The Raman spectra of MWCNTs displayed a lower I_D_/I_G_ ratio (1.06), indicative of higher graphitic ordering. In contrast, GNs showed a higher I_D_/I_G_ (1.45) and a lower I_2D_/I_G_ (0.42), consistent with a defect‐rich and strongly coupled multilayer structure. These values are typical of commercial MWCNTs and graphene‐based carbon materials. Upon varying the composition of carbon additives in the MgP_4_ composites, including different ratios of MWCNTs and GNs, systematic changes in the characteristic carbon‐related Raman bands were observed in MgP_4_/‐T2, T2G1, G2, and G2T1, respectively (Figure 3g). The resulting I_D_/I_G_ and I_2D_/I_G_ ratios reflected the relative contributions of MWCNTs and GNs, in agreement with the designed carbon mixing ratios. Furthermore, the spatial variation of these Raman ratios corresponded well with the local carbon composition, indicating a relatively uniform dispersion of CNTs and GNs. Combined with the homogeneous Mg, P, and C distributions observed by EDS mapping, these results demonstrated effective mixing of MgP_4_ with both carbon components while preserving their intrinsic structural characteristics.

**FIGURE 3:**
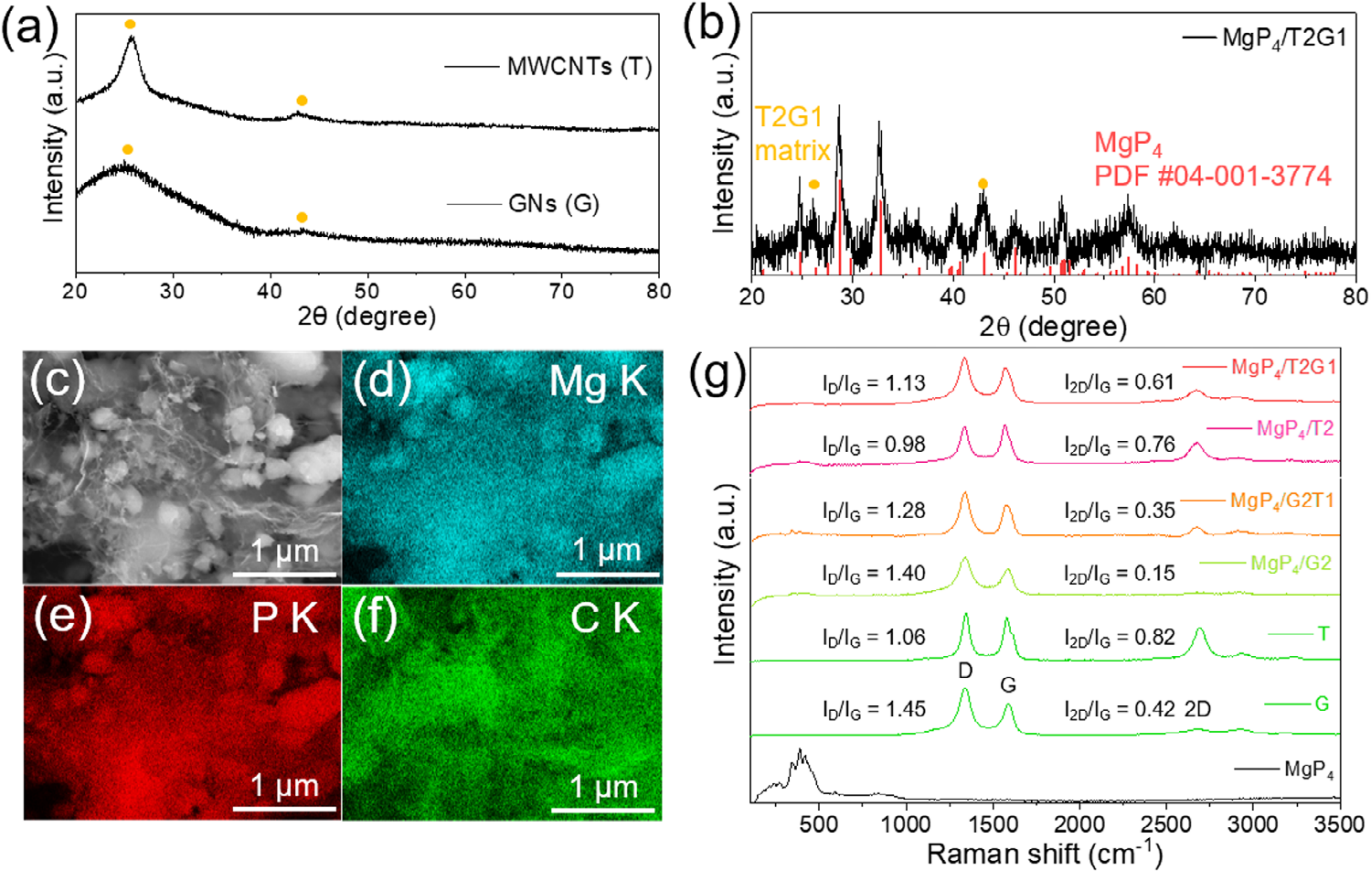
(a) XRD pattern of commercial MWCNTs and GNs. (b) XRD pattern, (c) SEM image, and corresponding EDS mapping images of (d) Mg K, (e) P K, and (f) C K of as‐synthesized MgP_4_/T2G1 composites. (g) Raman spectrum of carbon components (GNs and MWCNTs), MgP_4_, and their corresponding composites (MgP_4_/G2, MgP_4_/G2T1, MgP_4_/T2, MgP_4_/T2G1).

To further examine the dispersion state and structural evolution of carbonaceous matrix, SEM and TEM analysis were conducted. Commercial pristine MWCNTs and GNs powders exhibited agglomerated morphologies (Figure S4a,e), and even after manually mixing 20 wt.% of each carbon material with MgP_4_, micron‐sized carbon agglomerates persisted (Figure S4b,f). These agglomerates disappeared in the MgP_4_/T2 composites when a HEMM process was applied at a low rotation speed of 150 rpm (Figure S4c). The HR‐TEM image of MgP_4_/T2 composite revealed a few nanometer‐thick graphitic carbon layers on the particle surface (Figure S4d). These layers were attributed to the fragmentation, collapse, and subsequent reconstruction of MWCNTs into graphitic carbon during the HEMM process [44]. This observation indicated that the introduced MWCNTs were uniformly distributed and effectively anchored on the MgP_4_ particles surfaces. In contrast, agglomerates of GNs were still observed in the MgP_4_/G2 composite even after applying the same HEMM process (Figure S4g). This difference can be attributed to the 2D morphology and higher mechanical robustness of GNs, which render them less susceptible to fragmentation and surface anchoring during milling. Consequently, TEM image revealed heterogeneous regions with locally exposed particle surfaces, indicating non‐uniform carbon coverage within the composite (Figure S4h). Upon introducing an additional 10 wt.% of complementary carbon component followed by a second milling step to form a hybrid CNT‐G matrix, MgP_4_/‐T2G1 and G2T1 exhibited distinct microstructures. Low‐magnification SEM images showed no noticeable agglomerates in MgP_4_/T2G1, whereas MgP_4_/G2T1 still contained visible G agglomerates (Figure S5a,c). TEM images further revealed that MgP_4_/T2G1 formed a relatively well‐integrated dual‐layer carbon structure, consisting of an inner graphitic layer closely coupled with a thin outer amorphous carbon layer. In contrast, MgP_4_/G2T1 exhibited discontinuous carbon domains with interfacial voids, suggesting weaker carbon integration (Figure S5b,d). Therefore, the introduction of a hybrid CNT‐G matrix in MgP_4_/T2G1 is expected to promote more uniform carbon coverage and improved interfacial connectivity compared to the G2T1 configuration. To validate these potential benefits of MgP_4_/T2G1, a comparative electrochemical evaluation was performed.

The optimized MgP_4_/T2G1 electrode exhibited high initial discharge and charge capacities of 1673.3 and 1309 mAh g^−1^, respectively, corresponding to an ICE of 78.2% (Figure 4a). Benefiting from the optimized carbon matrix, the MgP_4_/T2G1 electrode delivered an enhanced reversible capacity compared to the pristine MgP_4_ electrode (Figure S3a,b). However, noticeable capacity fading was still observed, with a low capacity retention of 66% after 30 cycles, suggesting that significant volume changes of MgP_4_ particles persist even within the carbon matrix (Figure S3b). To improve the cycle retention of the MgP_4_/T2G1 electrode, a narrowed cutoff potential window was employed. This approach has been reported as an effective strategy for alloy‐type anodes because the narrowed cutoff potential window reduces the extent of sodiation/desodiation reactions and the associated volume variation of MgP_4_, which is expected to mitigate structural degradation and enhance cycling stability [45, 46] In this protocol, the cells were initially cycled for three cycles within a voltage window of 0.01–3.0 V vs. Na/Na^+^, followed by subsequent cycling within a narrowed window of 0.1–1.5 V vs. Na/Na^+^ (Figure 4a). Although this protocol resulted in a reduced reversible capacity of ∼1000 mAh g^−1^, it markedly improved cycling stability, delivering 986.3 mAh g^−1^ with 98.6% cycle retention over 50 cycles (Figure 4b). This value is 4.6 times higher than that of commercial HC electrode (216.3 mAh g^−1^), demonstrating the synergistic effect of the hybrid carbon matrix and cutoff potential control. Given the improved cycling stability, all subsequent electrochemical evaluations were conducted within the narrowed potential window unless otherwise specified. For comparison, even when pure red P was pre‐milled under identical conditions used for MgP_4_ synthesis and subsequently mixed with the same T2G1 hybrid carbon matrix (denoted as red P/T2G1), the resulting electrode showed a much lower reversible capacity of 498.1 mAh g^−1^ and a capacity retention of 77.7% over 50 cycles. These results indicate that the superior performance of MgP_4_/T2G1 cannot be attributed solely to the hybrid carbon matrix. Considering the reaction mechanism revealed by XANES analysis (Figure 2e), the enhanced electrochemical performance is likely associated with the finely dispersed Mg^0^/P composite generated through the initial conversion of MgP_4_, which provides a more favorable electrochemical environment than that obtained from red P alone. In the rate capability test (Figure 4c), the pristine MgP_4_ electrode was excluded due to its poor cycling stability, which prevented reliable capacity evaluation at varying current densities. The MgP_4_/T2G1 electrode delivered reversible capacities of 1139.4, 1097.6, 1024.9, 920.6, 690.8, and 530.0 mAh g^−1^ at current densities of 50, 100, 250, 500, 1000, and 2000 mA g^−1^, respectively. Compared to commercial HC, this capacity is 6.2 times higher at 50 mA g^−1^ and this difference increases to 14.8 times at 2000 mA g^−1^, indicating a more pronounced advantage under high rate conditions. Furthermore, the capacity retention from 50 to 2000 mA g^−1^ was 46.5% for MgP_4_/T2G1, compared to 36.0% for P/T2G1, further demonstrating the beneficial role of the Mg^0^‐derived activated composite formed from MgP_4_ in sustaining high rate electrochemical performance.

**FIGURE 4:**
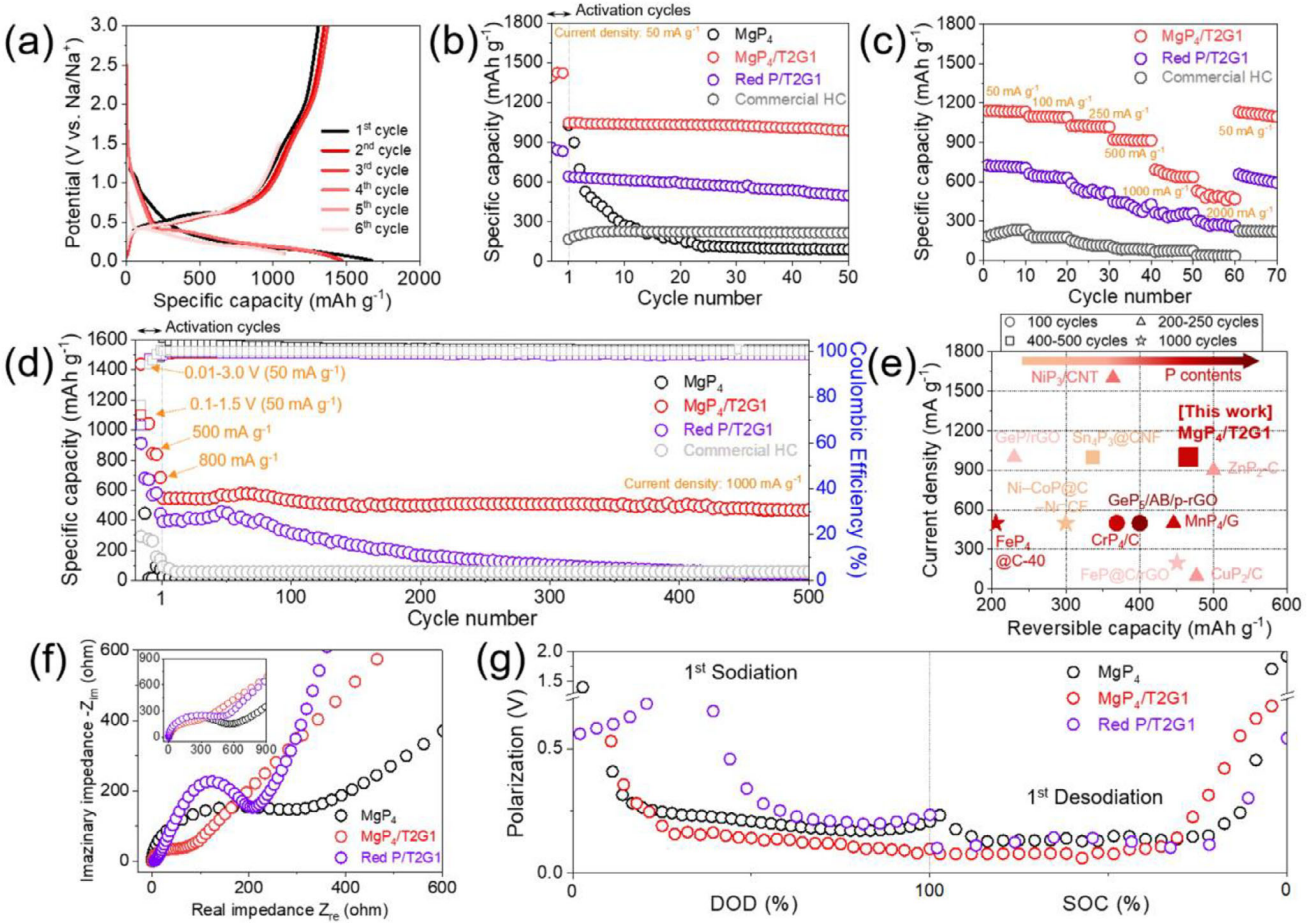
(a) Galvanostatic discharge/charge voltage profiles of MgP_4_/T2G1 electrode for the 1–3 cycles (0.01–3.0 V vs. Na/Na^+^) and 4–6 cycles (0.1–1.5 V vs. Na/Na^+^). (b) Cycle performance tested at 50 mA g^−1^, (c) rate capability, and (d) long‐term cycle performance tested at 1000 mA g^−1^ for SIBs. (e) Comparison of the electrochemical performance with state‐of‐the‐art phosphide anodes for SIBs. (f) Nyquist plots after 100 cycles in (d) (inset in (f) before cycle). (g) Measured polarizations from GITT profiles during first discharge and charge processes. The used electrolyte was 1.0 M NaClO_4_ in EC/DMC (1:1, v/v) with 5 vol.% FEC.

To evaluate long‐term cycle stability at a high current density of 1000 mA g^−1^, the electrodes were pre‐activated by cycling for 5 cycles each at 50, 500, and 800 mA g^−1^, followed by continuous cycling at 1000 mA g^−1^ (Figure 4d). The pristine MgP_4_ electrode exhibited rapid capacity degradation, with its reversible capacity becoming negligible within the first 10 cycles under high‐rate conditions. Commercial HC delivered only 55.9 mAh g^−1^ at 1000 mA g^−1^ due to sluggish Na^+^ ion diffusion associated with its insertion mechanism [47, 48]. The red P/T2G1 electrode initially delivered 435.6 mAh g^−1^ at 1000 mA g^−1^, but the capacity gradually faded and became practically negligible after 300 cycles. In contrast, MgP_4_/T2G1 electrode delivered an initial reversible capacity of 549.1 mAh g^−1^ at 1000 mA g^−1^ and maintained 468.5 mAh g^−1^ over 500 cycles, corresponding to a high capacity retention of 85.3%. The excellent long‐term cycling stability demonstrated that MgP_4_/T2G1 delivered high reversible capacity with robust performance at elevated current densities. Despite differences in experimental conditions among studies, the electrochemical performance of MgP_4_/T2G1 compares favorably with previously reported metal phosphide anodes for SIBs, particularly in terms of reversible capacity retention under high current density operation (Figure 4e and Table S2).

To gain insights into the origin of the superior electrochemical performance of MgP_4_/T2G1 electrode compared to the other electrodes, electrochemical impedance spectroscopy (EIS) was applied. The Nyquist plots of pristine MgP_4_, MgP_4_/T2G1, and red P/T2G1 electrodes exhibited different charge transfer resistance (R_ct_) values of 505, 350, and 440 ohm, respectively, in the high‐frequency region, whereas similar Warburg slopes were observed in the low‐frequency region, suggesting comparable Na^+^ ion diffusivity (inset in Figure 4f). After 100 cycles at 1000 mA g^−1^, all electrodes exhibited lower R_ct_ values than in the pristine state, and these behaviors are similar to previous reports on red P and phosphide‐based high capacity anodes and may be associated with electrochemical activation during cycling [31, 32, 49, 50]. Notably, MgP_4_ electrode exhibited the largest R_ct_, whereas MgP_4_/T2G1 electrode maintained the lowest R_ct_ among the electrodes, suggesting that the hybrid carbon matrix enabled more efficient charge transfer and better maintained conductive pathways during repeated sodiation/desodiation reactions (Figure 4f). To further quantify the polarization associated with electrochemical reactions and phase transitions during sodiation/desodiation, the Galvanostatic intermittent titration technique (GITT) was employed during the first cycle (Figure 4g). In the GITT measurements, a constant current of 50 mA g^−1^ was applied for 1 h, followed by a 3 h rest period under open circuit conditions (Figure S6). As shown in Figure 4g, the MgP_4_ electrode exhibited a polarization of 0.23 V at an initial depth of discharge (DOD) of 30%, which gradually decreased to 0.17 V up to a DOD of 80% during sodiation, followed by a slight increase to 0.21 V near full sodiation. During desodiation, the polarization remained around 0.14 V until the state of charge (SOC) of 15%, after which it increased sharply. By contrast, the red P/T2G1 electrode showed a rapid increase in polarization with increasing initial DOD, reaching as high as 0.68 V. Although the polarization decreased beyond a DOD of 45%, it remained higher than that of the MgP_4_ electrode during sodiation, while exhibiting comparable polarization during desodiation. Notably, the MgP_4_/T2G1 electrode showed polarization behavior similar to that of MgP_4_ at the early stage, decreasing to 0.16 V by a DOD of 30%. However, the polarization continuously decreased to ∼0.09 V near full sodiation, representing the lowest value among the three electrodes. Moreover, during desodiation, MgP_4_/T2G1 electrode maintained the lowest polarization up to an SOC of 70%. This demonstrates that the conductive hybrid T2G1 matrix, in combination with the intrinsic MgP_4_ phase, efficiently mitigated polarization by improving charge transport and reaction kinetics, highlighting the critical role of the carbon matrix in governing electrochemical behavior.

### 2.4 Unraveling Matrix‐Dependent Differences Between T2G1 and G2T1

To isolate the effect of the carbon matrix, the MgP_4_/G2T1 electrode was comparatively evaluated against the MgP_4_/T2G1 electrode. The MgP_4_/G2T1 electrode delivered a sodiation capacity of 1466 mAh g^−1^ with a lower ICE of 73.6%, corresponding to a reversible desodiation capacity of 1079 mAh g^−1^, both of which are inferior to those of MgP_4_/T2G1 (Figure 4a,b and Figure S7a,b). In rate capability tests, MgP_4_/T2G1 retained 46.5% of its capacity when the current density increased from 50 to 2000 mA g^−1^, whereas MgP_4_/G2T1 retained only ∼22%, indicating significantly poorer high‐rate performance, accompanied by inferior long‐term cycling stability at 1000 mA g^−1^ (Figure S7c,d). To elucidate the origin of the difference in rate capability, kinetic analysis was conducted based on cyclic voltammetry (CV) measurements at scan rates ranging from 0.1 to 2.0 mV s^−1^ (Figure S8a–c). The current response at a given potential was analyzed using the relationship i(V) = k_1_v + k_2_v^1/2^, where k_1_v represents the capacitive contribution, and k_2_v^1/2^ corresponds to diffusion‐controlled processes [51]. The normalized contributions revealed that both MgP_4_/T2G1 and MgP_4_/G2T1 exhibited higher pseudocapacitive contributions than pristine MgP_4_ (Figure S8d–f). Notably, the T2G1 matrix delivered a larger pseudocapacitive contribution than the G2T1 counterpart, indicating more favorable surface‐controlled kinetics that facilitated rapid charge storage at high rates.

To further assess the generality of the matrix‐dependent behavior observed in the MgP_4_ system, MnP_4_ was introduced alongside red P and MgP_4_ while maintaining identical T2G1 and G2T1 matrices for direct comparison. MnP_4_ was synthesized via the previously reported HEMM method [31], enabling systematic evaluation of intrinsic active material effects independent of matrix architecture. When evaluated at a current density of 50 mA g^−1^, the MnP_4_/T2G1 electrode exhibited initial discharge and charge capacities of 1068 and 897 mAh g^−1^, respectively, with an ICE of 83.9% (Figure S9a). Within a narrowed cutoff potential window of 0.1–1.5 V, a reversible capacity of ∼900 mAh g^−1^ was maintained, which is higher than that of red P/T2G1, but lower than that of MgP_4_/T2G1 (Figure 4b and Figure S9b). Interestingly, replacing T2G1 with G2T1 resulted in a substantial reduction in reversible capacity (∼300 mAh g^−1^ lower), further reinforcing the matrix‐dependent performance trend observed in MgP_4_ systems. During long‐term cycling at 1000 mA g^−1^, MnP_4_/G2T1 exhibited gradual capacity decay after 50 cycles, whereas MnP_4_/T2G1 retained ∼93% of its capacity over 300 cycles (Figure S9c). Post‐cycling EIS and GITT measurements revealed higher charge‐transfer resistance and larger polarization for MnP_4_/T2G1 than for MgP_4_/T2G1, indicating slower reaction kinetics in the MnP_4_ system (Figure S10 and Table S3). These results suggest that the superior performance of MgP_4_/T2G1 arises from the combined effect of intrinsically faster reaction kinetics of MgP_4_ and the conductive T2G1 matrix. Notably, this matrix‐dependent behavior was not limited to SIB systems. Given that MgP_4_ is also electrochemically active in LIBs, analogous comparisons using G2T1 and T2G1 matrices revealed that the T2G1 framework consistently delivered higher capacity and superior rate capability, confirming its general effectiveness across alkali‐ion battery systems (Figure S11).

To elucidate the origin of matrix‐dependent electrochemical behaviors among the MgP_4_, MgP_4_/T2G1, and MgP_4_/G2T1, the charge transport characteristics within the electrodes were investigated using atomic force microscopy (AFM) and scanning spreading resistance microscopy (SSRM) analyses. The cross‐section of the electrodes was prepared via ion milling, and the spatial distribution of electrical resistance was obtained to understand how the carbon matrix structure governs charge transport. Since the electrodes were prepared without conductive agent of Super P and CMC acts as an electronically insulating binder, the contrast in SSRM images primarily reflects the distribution and connectivity of the hybrid carbon network structures within the electrode [52, 53, 54]. Cross‐sectional AFM topography and SSRM mapping image of pristine MgP_4_ electrode (Figure 5a) revealed a large fraction of high resistance regions, indicating poor electrical conductivity and discontinuous conductive pathways. While the incorporation of conductive carbon matrices reduced these resistive domains, distinct differences remained depending on the matrix configuration. MgP_4_/G2T1 electrode exhibited narrower high resistance regions compared to pristine MgP_4_, yet still contained localized clusters of resistive domains, suggesting incomplete and discontinuous conductive pathways (Figure 5b). In contrast, MgP_4_/T2G1 exhibited the lowest overall resistance, with low resistance domains uniformly distributed across the electrode thickness (Figure 5c), indicating the formation of continuous and interconnected charge transport pathways rather than conduction limited to particle contact points.

**FIGURE 5:**
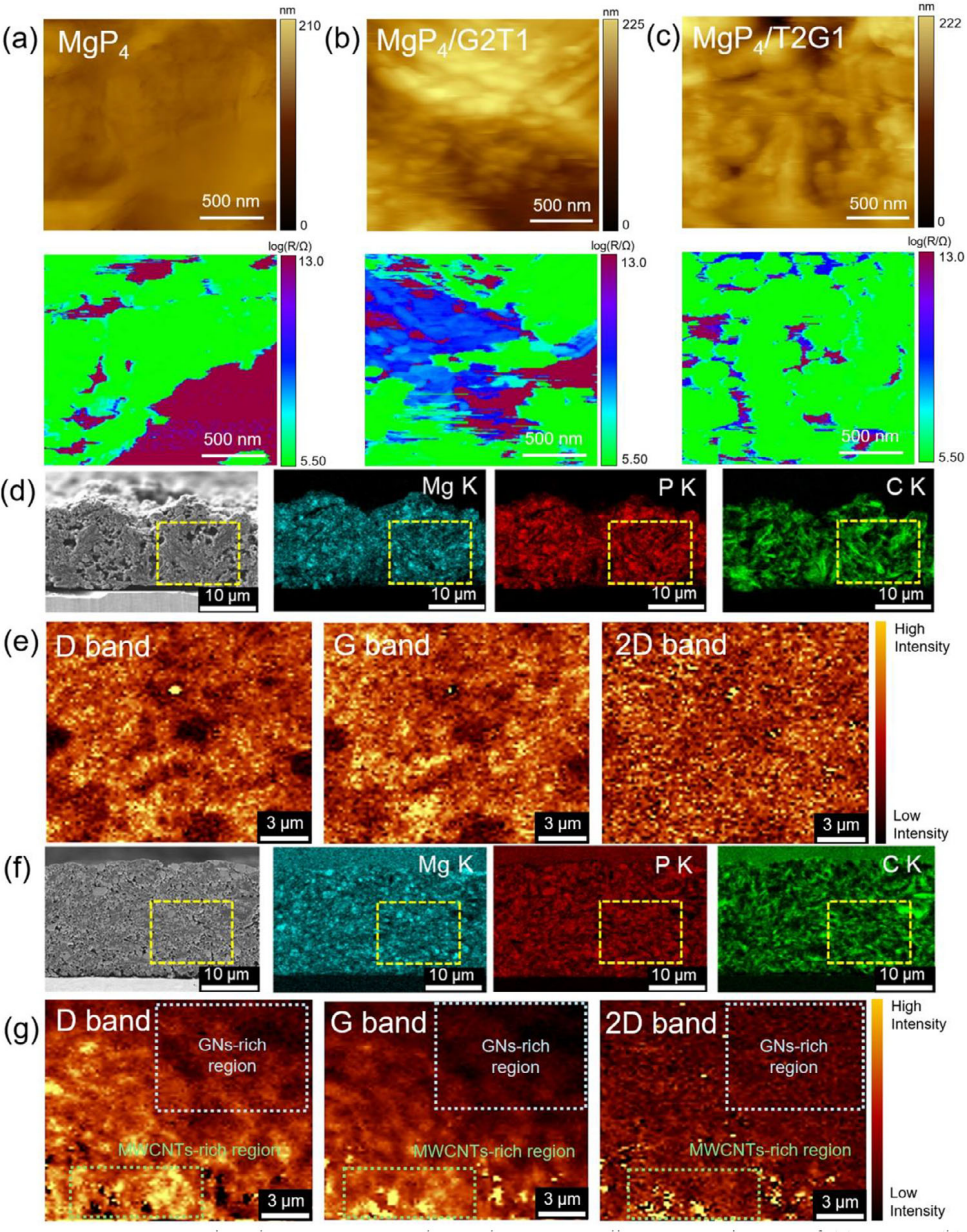
Cross sectional AFM topography and corresponding SSRM image of (a) MgP_4_, (b) MgP_4_/G2T1, (c) MgP_4_/T2G1 electrodes. Cross sectional SEM image and corresponding EDS mapping images of Mg K, P K, and C K, and Raman band mapping images of D, G, and 2D bands for (d,e) MgP_4_/T2G1 and (f,g) MgP_4_/G2T1 electrodes, respectively.

To further understand the structural origin of these differences, cross‐sectional EDS and Raman mapping analyses were carried out. To clearly distinguish between CNTs and GNs, electrodes were prepared without conductive CB (Super P) and subsequently analyzed after ion milling. EDS mapping revealed a uniform distribution of C along with Mg and P in both MgP_4_/T2G1 and MgP_4_/G2T1 electrodes (Figure 5d,f). However, Raman mapping, which distinguishes D, G, and 2D bands, revealed pronounced differences between the two composites. MgP_4_/T2G1 exhibited relatively uniform intensity distributions across all bands throughout the electrode area, indicating a well‐integrated hybrid carbon framework (Figure 5e). In contrast, MgP_4_/G2T1 showed significant spatial variations in the D, G, and particularly 2D band intensities (Figure 3g), suggesting heterogeneous distributions of GN‐rich and MWCNT‐rich regions and a less integrated carbon network (Figure 5g).

These structural differences likely arose from the distinct assembly sequences of the hybrid matrices. In the T2G1 configuration, the initially introduced CNTs may form continuous conductive bridges on the active material surface, while also serving as a physical spacer and anchoring framework for the subsequently added GNs. Such a hierarchical structure can suppress graphene restacking and promote the formation of a more homogeneous hybrid carbon network, which is expected to facilitate efficient charge percolation throughout the electrode [55, 56]. In contrast, when GNs are introduced first in the G2T1 configuration, strong intersheet interactions may lead to localized graphene‐rich aggregates (Figure S4g), limiting the uniform incorporation of subsequently added CNTs and resulting in less integrated conductive pathways. The more interconnected CNT–GN hybrid framework in T2G1 matrix is therefore expected to enable more efficient electron transport and reduced polarization, thereby contributing to the enhanced rate capability of MgP_4_/T2G1 electrodes.

Beyond improved charge transport, the interconnected carbon framework is also expected to enhance mechanical durability during repeated cycling. To verify this, *ex‐situ* SEM analysis was performed (Figure S12). The MgP_4_ electrode cycled at 1000 mA g^−1^ showed severe structural degradation within the initial 10 cycles (Figure 4d), including extensive cracking and delamination from the current collector (Figure S12a,b). The electrode thickness increased by ∼250%, likely due to the formation of thick SEI layers and particle agglomeration, which generated large voids and disrupted electrical pathways (Figure S12c,d). In contrast, MgP_4_/T2G1 electrode exhibited only minor cracking even after 500 cycles at 1000 mA g^−1^, without noticeable delamination or severe particle agglomeration (Figure S12e,f). Cross‐sectional SEM images revealed that the electrode remained densely packed, with volume expansion limited to ∼10% despite prolonged cycling (Figure S12g,h), demonstrating superior mechanical integrity. High‐magnification SEM analysis before and after cycling further confirmed the role of the hybrid matrix in suppressing particle agglomeration. The pristine MgP_4_ electrode initially contained well‐dispersed CB, but after cycling, thick SEI layers and large agglomerates were formed (Figure S13a,b). In contrast, MgP_4_/T2G1 electrodes maintained a robust conductive framework that bridged active materials, while the MWCNTs–GNs network remained intact even after prolonged cycling (Figure S13c,d), highlighting its critical role in enhancing electrode durability.

Overall, these results demonstrate that the assembly sequence of hybrid carbon matrices governs not only electronic connectivity but also mechanical robustness. The well‐integrated T2G1 network simultaneously enables efficient charge transport, mitigates volume expansion, and preserves electrode integrity, thereby unlocking the intrinsic high‐capacity potential of MgP_4_ through rational composite design (Scheme 2).

**SCHEME 2:**
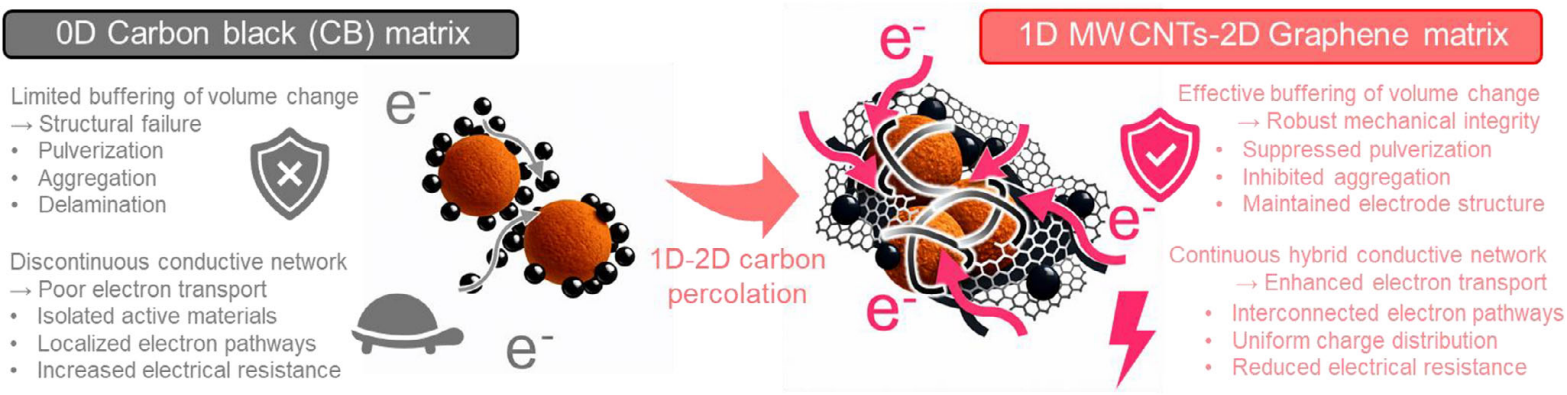
Schematic illustration of origin of the differences in electrochemical performance between MgP_4_ and MgP_4_/T2G1 electrodes.

### 2.5 Electrochemical Performance of MgP_4_/T2G1@HC Composite

To evaluate the practical applicability of the MgP_4_/T2G1 composite for high‐energy density SIBs, the composite was integrated into a commercial HC matrix (MgP_4_/T2G1@HC), and its electrochemical performance was investigated. The commercial HC was characterized by XRD and Raman spectroscopy, showing a typical diffraction pattern and characteristic D and G bands, indicative of a typical turbostratic and disordered carbon structure (Figure S14a,b) [57]. SEM image of commercial HC revealed platelet‐like particles with sizes of approximately sub‐10 µm (Figure S14c).

The MgP_4_/T2G1@HC composite was prepared via soft ball milling at 100 rpm for 1 h with a weight ratio of 3:7 (HC:MgP_4_/T2G1). The resulting electrode exhibited a dense and well‐packed morphology with minimal macropores (Figure S14d), suggesting effective particle bridging and void filling during electrode fabrication and calendaring processes. Before electrochemical evaluation, the electrolyte conditions were optimized to ensure stable operation of the commercial HC anode. By adjusting the electrolyte composition to 1.3 M NaClO_4_ in EC:DMC (1:1, v/v) with 2 vol.% FEC, the commercial HC electrode exhibited the highest reversibility, delivering a reversible capacity of 263.5 mAh g^−1^ over 100 cycles at 50 mA g^−1^ (Figure S15a,b).

Using the optimized electrolyte, the MgP_4_/T2G1@HC electrode delivered a high reversible capacity of 547.7 mAh g^−1^ with an ICE of 78.2% at 50 mA g^−1^ (Figure 6a). After activation and stepwise current increase, long‐term cycling at a high current density of 1000 mA g^−1^ demonstrated stable performance, retaining 146.5 mAh g^−1^ after 2000 cycles, which corresponds to approximately 2.6 times higher capacity than that of a commercial HC electrode, outperforming previously reported hard carbon anodes in both carbonate‐ and ether‐based electrolytes (Figure 6b,c and Table S4). Notably, this enhanced performance was achieved via a simple physical blending strategy, highlighting the practical compatibility of the MgP_4_/T2G1 structure with conventional hard carbon hosts.

**FIGURE 6:**
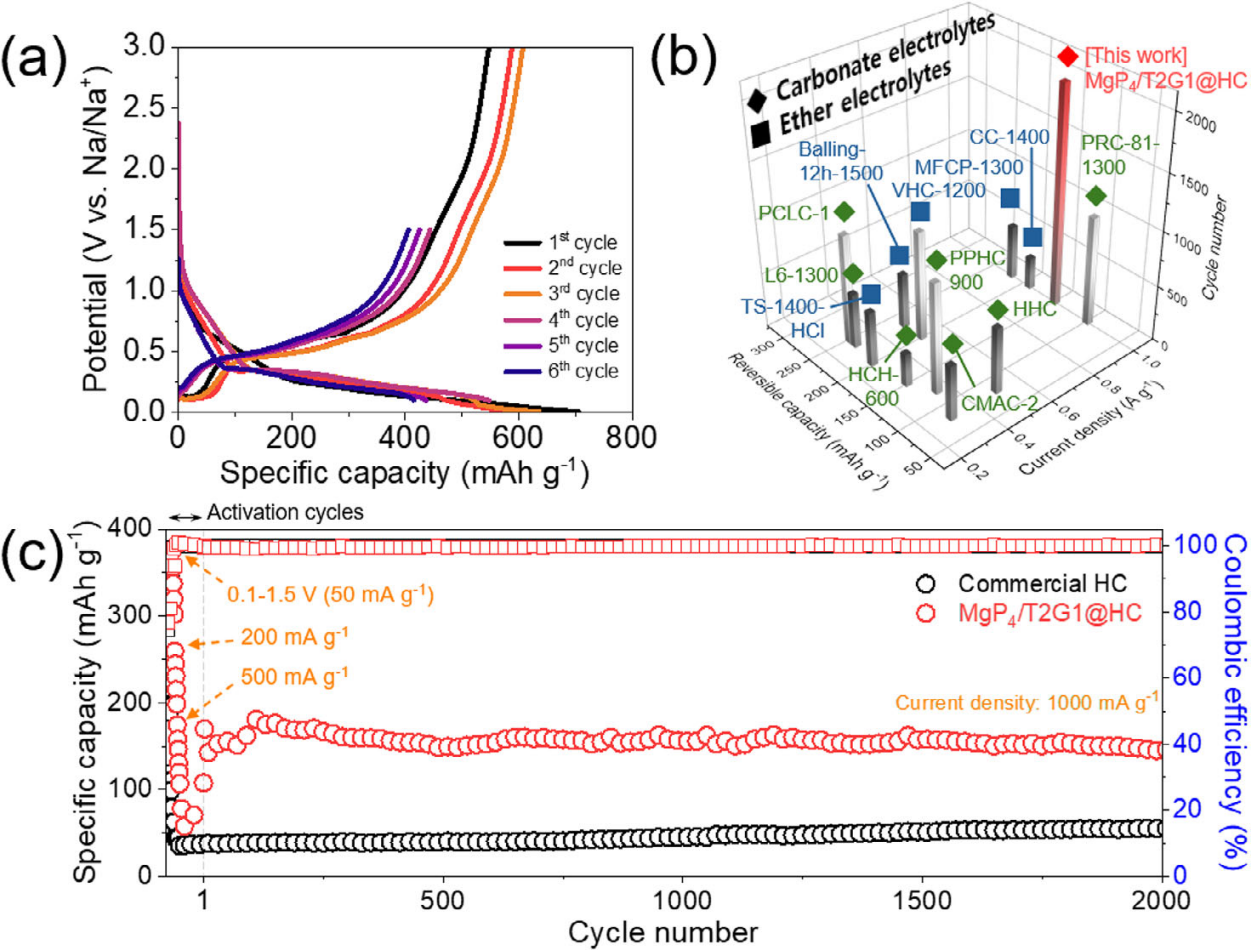
(a) Galvanostatic discharge/charge voltage profiles of MgP_4_/T2G1@HC electrode tested at a current density of 50 mA g^−1^. (b) Comparison of the electrochemical performance with state‐of‐the‐art hard carbon anodes for SIBs. (c) Long‐term cycle performance of commercial HC and MgP_4_/T2G1@HC electrodes tested at 1000 mA g^−1^ for SIBs. The used electrolyte was 1.3 M NaClO_4_ in EC/DMC (1:1, v/v) with 2 vol.% FEC.

To evaluate the practical applicability of MgP_4_/T2G1@HC electrode for full cell applications, a full cell was assembled using commercial Na_4_Fe_3_(PO_4_)_2_P_2_O_7_ (NFPP) as the cathode electrode (Figure 7a and Figure S16). NFPP electrode was fabricated with a mass loading of ∼3.5 mg cm^−2^. The negative to positive electrode charge ratio (N/P ratio) of NFPP || MgP_4_/T2G1@HC full cell was controlled at ∼1.1. Prior to full cell assembly, both the cathode and anode electrodes were pre‐cycled at a current density of 100 mA g^−1^ for a formation cycle. NFPP || MgP_4_/T2G1@HC full cell was galvanostatically cycled at a high current density of 10 C (1170 mA g^−1^) for 1000 cycles after activation at 0.1 C for 2 cycle and 5 C for 3 cycles. The full cell delivered an initial charge and discharge capacities of 102.8 mAh g^−1^ and 89.3 mAh g^−1^, respectively, at 0.1 C (11.7 mA g^−1^), corresponding to an ICE of 86.9% (Figure 7b). Subsequently, the full cell exhibited an initial discharge capacity of approximately 73.4 mAh g^−1^ at a high current density of 10 C and maintained a discharge capacity of 55.1 mAh g^−1^ after 1000 cycles, corresponding to a capacity retention of 75.1% (Figure 7c). These results demonstrate the excellent long‐term cycling stability of the NFPP || MgP_4_/T2G1@HC full cell under high‐rate operating conditions and further confirm the practical feasibility of the MgP_4_/T2G1@HC anode for high‐energy SIBs. By combining the high capacity of MgP_4_/T2G1 with the structural stability and cycling durability of HC, the MgP_4_/T2G1@HC composite provides a promising anode design strategy for high‐energy SIBs, analogous to the Si/graphite composite concept widely adopted in commercial LIBs.

**FIGURE 7:**
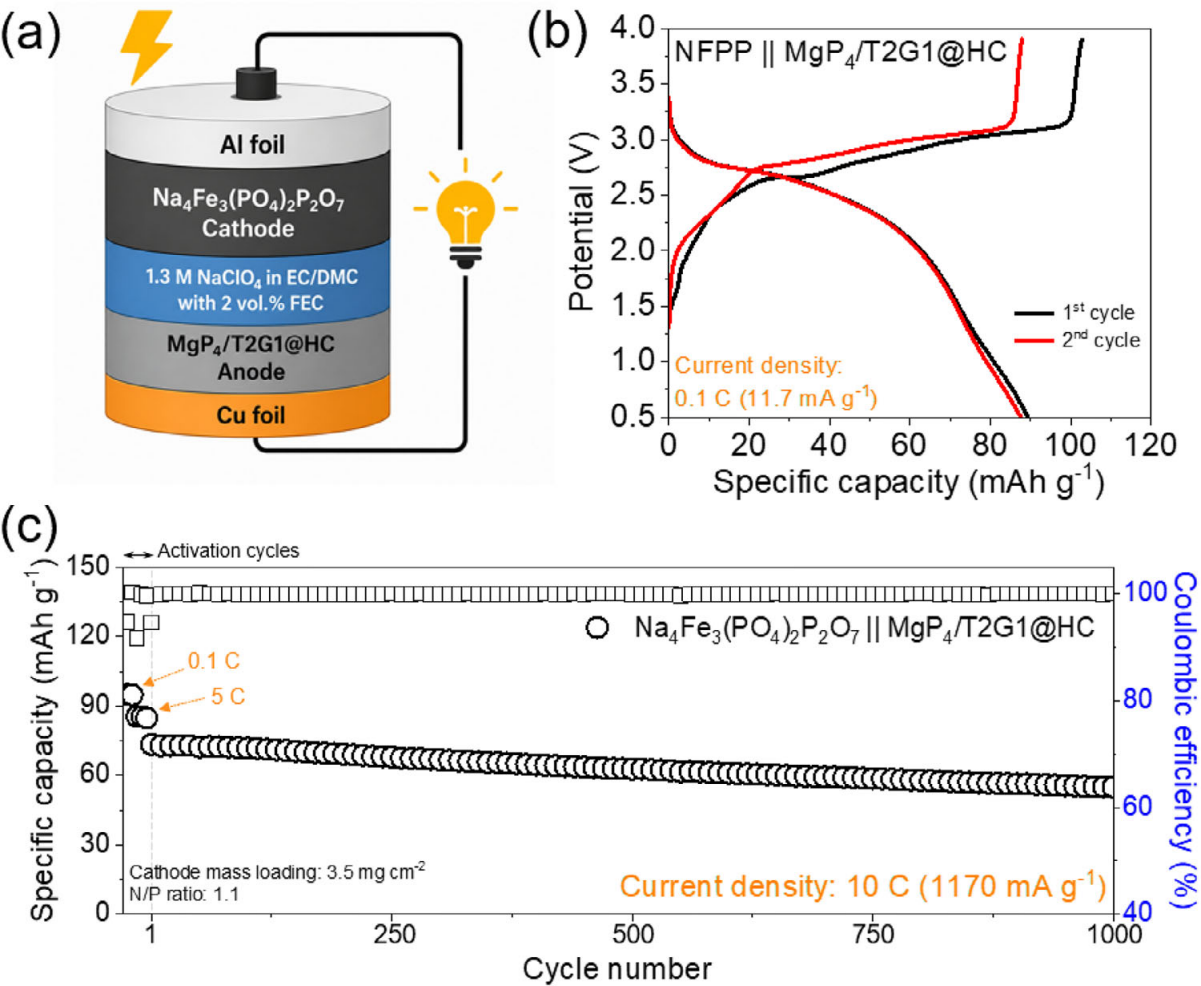
(a) Schematic illustration of Na_4_Fe_3_(PO_4_)_2_P_2_O_7_ (NFPP) || MgP_4_/T2G1@HC full cell configuration, (b) galvanostatic charge/discharge voltage profiles at 0.1 C, and (c) long‐term cycle performance of the full cell at 10 C after activation at 0.1 C for 2 cycles and 5 C for 3 cycles. The used electrolyte was 1.3 M NaClO_4_ in EC/DMC (1:1, v/v) with 2 vol.% FEC.

## 3 Conclusion

In summary, we introduce MgP_4_, a P‐rich alkaline‐earth metal phosphide that expands beyond conventional transition‐metal phosphides as a high capacity anode for SIBs. MgP_4_ particles were synthesized within 2 h via a facile and scalable HEMM process under ambient conditions. When evaluated in both sodium‐ and lithium‐ion systems, MgP_4_ exhibited robust charge‐storage capability. *Ex situ* XRD and XANES analyses indicated that the initial sodiation proceeds through a conversion reaction, followed by reversible alloying/dealloying reactions involving the P component. To enhance the electrochemical performance, we employed a carbon‐based composite strategy and demonstrated that both the configuration and assembly sequence of the carbon matrix played an important role. The optimized hybrid T2G1 matrix served a continuous percolation network and mechanically resilient framework, as confirmed by cross‐sectional SSRM and Raman mapping, as well as ex situ SEM. This rational matrix engineering enabled stable high‐rate cycling of MgP_4_/T2G1 in SIBs, delivering 468.5 mAh g^−1^ with 83.5% capacity retention over 500 cycles at 1000 mA g^−1^. To demonstrate practical applicability, 30 wt.% MgP_4_/T2G1 was further integrated into a commercial HC host via simple ball milling. The resulting MgP_4_/T2G1@HC composite exhibited long‐term cycling stability over 2000 cycles at 1000 mA g^−1^, retaining 146.5 mAh g^−1^, which corresponds to ∼2.6 times higher capacity than that of HC. Analogous to graphite/Si composite strategies in LIBs, the MgP_4_/T2G1@HC provided a scalable pathway for translating high‐capacity alloying chemistries into practical, high‐energy SIB anodes.

## 4 Experimental Section

### 4.1 Synthesis of MgP_4_ and MnP_4_ Nanoparticles

MgP_4_ nanoparticles were synthesized via a high‐energy mechanochemical milling (HEMM) using a planetary ball mill (Pulverisette 6, FRITSCH). Commercial magnesium (Mg, 98%, Sigma‐Aldrich) and red phosphorus (P, 98%, Thermo Scientific) powders were used without further purification. Stoichiometric amounts of Mg and P with a molar ratio of 1:4 were loaded into a hardened steel vial (80 cm^3^) containing 5 mm diameter of hardened steel balls and sealed in an argon‐filled glove box (O_2_< 0.1 ppm, H_2_O < 0.1 ppm). The HEMM process was performed at 400 rpm for 2 h with a ball‐to‐powder weight ratio of 50:1 at room temperature. (Caution: Since residual Mg nanopowder may be pyrophoric upon exposure to air, phase analysis using an air‐tight sample holder is recommended to confirm reaction completion.) MnP_4_ nanoparticles were synthesized using the same procedure, except manganese powder (Mn, 99.95%, Thermo Scientific) was used and milling was extended to 28 h.

### 4.2 Fabrication of MgP_4_‐Based, Hybrid Carbon Composites

MgP_4_‐based composites were prepared via a two‐step HEMM process under an argon atmosphere. In the first step, MgP_4_ was milled with multi‐walled carbon nanotubes (MWCNTs, 99%, Carbon Nano‐material Technology Co.,Ltd.) or graphene nanosheets (GNs, 97.8%, ANGSTRON MATERIALS, INC) at 150 rpm for 1.5 h. In the second step, the complementary carbon component was introduced and milled under identical conditions to form a hybrid CNT/G matrix. Carbon components are denoted as C (super P carbon black), T (MWCNTs) and G (GNs), and the numbers indicate their weight percentages (e.g., T2G1 corresponds to 20 wt.% CNTs followed by 10 wt.% GNs). The sequence reflects the order of incorporation during the two‐step milling process. MnP_4_‐based composites were prepared using the same protocol. For comparison, red P‐based composites were fabricated by pre‐milling red P under identical conditions used for MgP_4_ synthesis, followed by the same two‐step composite process.

### 4.3 Fabrication of MgP_4_/T2G1@Hard Carbon (HC) Composites

Commercial HC (MTI Co. Ltd., South Korea) and MgP_4_/T2G1 composites were mixed at a weight ratio of 70:30 and ball‐milled at 100 rpm for 1 h (ball‐to‐powder ratio 20:1) under an argon atmosphere to obtain a homogeneous composite.

### 4.4 Electrolyte Preparation

Ethylene carbonate (EC, 99%, Sigma–Aldrich) and dimethyl carbonate (DMC, 99%, Sigma–Aldrich) were mixed at a 1:1 volume ratio and then stored over molecular sieves (> 3 days) before use. NaClO_4_ (99%, Sigma–Aldrich) salt was dried overnight at 100 °C under a glass vacuum oven (G‐300, Buchi) before use and dissolved in the EC:DMC mixture to obtain concentrations of 1.3 M and 1.5 M. Subsequently, fluoroethylene carbonate (FEC, 99%, Sigma–Aldrich) was added as an additive (2–5 vol.%). The electrolyte was stirred at 40 °C for 24 h under an inert argon atmosphere.

### 4.5 Materials Characterization

Synchrotron x‐ray powder diffraction (SXPD) patterns of the as‐synthesized MgP_4_ nanoparticles were obtained at the 9B high‐resolution powder x‐ray diffraction (HRPD) beamline of the Pohang Accelerator Laboratory (PAL, Korea). The storage ring had an acceleration voltage of 2.5 GeV and a ring current of 330–360 mA. The incident beam was vertically collimated using a mirror and monochromatized to a wavelength of 1.5299 Å using a double‐crystal Si (111) monochromator. The diffraction patterns were collected in the 2θ scan mode with a step size of 0.02° and a step time of 13 s, ranging from 10° to 130°. The lattice parameters of the as‐synthesized MgP_4_ nanoparticles were calculated using the FullProf software. Conventional XRD measurements were conducted using x‐ray diffractometer (XRD, UltimaIV, Rigaku) with Cu Kα (*λ* = 1.5406 Å) radiation. Raman spectroscopy (NRS‐5100, JASCO) was conducted using a 532 nm laser (0.4 mW, 60 s exposure) to analyze carbon structures (D/G/2D bands) and to perform Raman mapping for evaluating the spatial distribution of carbon components. Cross‐sectional electrical resistance mapping was conducted using scanning spreading resistance microscopy (SSRM) based on atomic force microscopy (AFM, Cypher S, Oxford Instruments). To eliminate the influence of additional conductive carbon additives on the Raman mapping and SSRM analyses, electrodes consisting of 90 wt.% active materials (AM) and 10 wt.% carboxymethyl cellulose (CMC) binder were prepared without a conductive agent of Super P. Morphology of as‐synthesized powders and electrodes was observed by field‐emission scanning electron microscopy (FE‐SEM, JSM‐IT800SHL, JEOL) and transmission electron microscopy (TEM, JEM‐2100F, JEOL). For ex‐situ XRD and SEM analyses of the electrodes, the cycled coin cells were disassembled in an argon‐filled glove box and rinsed with DMC several times.

### 4.6 Electrochemical Measurements

Electrodes were prepared by mixing 70 wt.% AM, 15 wt.% Super P, and 15 wt.% of CMC binder to form a slurry, which was coated onto copper (Cu) foil. After room temperature drying, the electrodes were transferred to a vacuum oven and dried at 80 °C overnight. The calendaring process was performed properly to increase its electrode density by approximately 2–3 times, from an initial value of 1.2–1.8 g cm^−^^3^ and mass loading of active material was 1.0–1.5 mg cm^−2^. The commercial hard carbon electrodes were formulated by blending 94 wt.% active materials, 3 wt.% Super P and 3 wt.% CMC binder and the mass loading of active materials was controlled 2–2.5 mg cm^−2^. MgP_4_/T2G1@HC electrodes used a slurry formulation of 92:3:5 (AM:Super P:CMC) and the mass loading of AM was ∼2.0 mg cm^−2^. CR2032 coin cells were assembled in an argon‐filled glove box (O_2_< 0.1 ppm, H_2_O < 0.1 ppm) using sodium metal as counter/reference electrodes and glass fiber separators (Whatman). The used electrolytes were 1.0 M NaClO_4_ in a mixture of EC and DMC (1:1, v/v) with the addition of 5 vol% of FEC additive and 1.3 M NaClO_4_ in a mixture of EC and DMC (1:1, v/v) with the addition of 2 vol% of FEC additive. Lithium cells were also assembled similarly using Li metal as a counter/reference electrode and 1.0 M LiPF_6_ in a mixture of EC, DEC and dimethyl carbonate (DMC) (1:1:1, v/v) with an addition of 10 vol% of FEC. Galvanostatic cycling tests were conducted using a battery testing system (WBCS3000L, Wonatech, South Korea). For sodium‐ion cells, cutoff voltage was optimized by narrowing the voltage window from 0.01–3.0 V to 0.1‐1.5 V (vs. Na/Na^+^). Similarly, lithium‐ion cells were tested within voltage ranges of 0.01–3.0 V to 0.01–1.5 V (vs. Li/Li^+^). Electrochemical impedance spectroscopy (EIS) experiment was carried out over a frequency range of 100 kHz to 0.1 Hz with an AC amplitude of 5 mV using an impedance analyzer (Zive SP1, Wonatech, South Korea). Galvanostatic intermittent titration technique (GITT) measurements were performed at a current density of 50 mA g^−1^ with 1 h current pulse followed by 3 h relaxation in the voltage range of 0.01–3.0 V (vs. Na/Na^+^). Na_4_Fe_3_(PO_4_)_2_P_2_O_7_ (NFPP) || MgP_4_/T2G1@HC full cells were assembled using commercial NFPP (MTI Korea) as the cathode and MgP_4_/T2G1@HC as the anode. NFPP cathode was prepared by mixing 94 wt.% AM, 3 wt.% Super P, and 3 wt.% of polyvinylidene fluoride (PVDF) binder to form a slurry, which was coated onto aluminum (Al) foil. NFPP cathode was prepared with a mass loading of approximately 3.5 mg cm^−2^ to achieve an N/P ratio of 1.05–1.10, which was determined based on the reversible capacities obtained after precycling NFPP cathode and MgP4/T2G1@HC anode with voltage ranges of 4.0‐2.0 V vs. Na/Na^+^ and 0.1‐1.5 V vs. Na/Na^+^, respectively. The electrolyte consisted of 1.3 M NaClO_4_ dissolved in an EC/DMC (1:1, v/v) mixture with 2 vol.% FEC. Full cell electrochemical performance was evaluated at room temperature within a voltage window of 0.5–3.9 V.

## Author Contributions

**Sion Ha**: writing – original draft, methodology, investigation, data curation, validation, formal analysis, visualization. **Doyeon Lee**: methodology, validation, investigation. **Kyeong‐Ho Kim**: conceptualization, writing – review and editing, project administration, resources, funding acquisition, visualization, supervision. **Dong Won Kim**: validation, investigation. **Won‐Sik Kim**: conceptualization. **Seong‐Hyeon Hong**: funding acquisition, resources, validation. **Minkyu Lee**: validation, investigation, methodology. **Seong‐Hyeon Hong**: funding acquisition, resources, validation. **Kyeong‐Ho Kim**: conceptualization, writing – review and editing, project administration, resources, funding acquisition, visualization, supervision.

## Conflicts of Interest

The authors declare no conflicts of interest.

## Supporting information




**Supporting File**: advs76701‐sup‐0001‐SuppMat.doc.

## Data Availability

The data that support the findings of this study are available from the corresponding author upon reasonable request.
