# Supplementary material for: MgP4/CNT‐Graphene Embedded in Hard Carbon Matrix as a High‐Capacity Anode for Next‐Generation Sodium‐Ion Batteries
Source: Adv Sci (Weinh). 2026 Jul 17:e76701. Online ahead of print. doi: 10.1002/advs.76701 (PMC13379213; doi:10.1002/advs.76701)
Supplement: Supplementary file 1 — Supporting File: advs76701‐sup‐0001‐SuppMat.doc. [file ADVS-9999-e76701-s001.doc]

Copyright WILEY-VCH Verlag GmbH & Co. KGaA, 69469 Weinheim, Germany, 2025.

Supporting Information

**MgP_4_/CNT-Graphene Embedded in Hard Carbon Matrix as a High-Capacity Anode for Next-Generation Sodium-Ion Batteries**

Sion Ha, Doyeon Lee, Dong Won Kim, Won-Sik Kim, Minkyu Lee, Seong-Hyeong Hong, and Kyeong-Ho Kim*


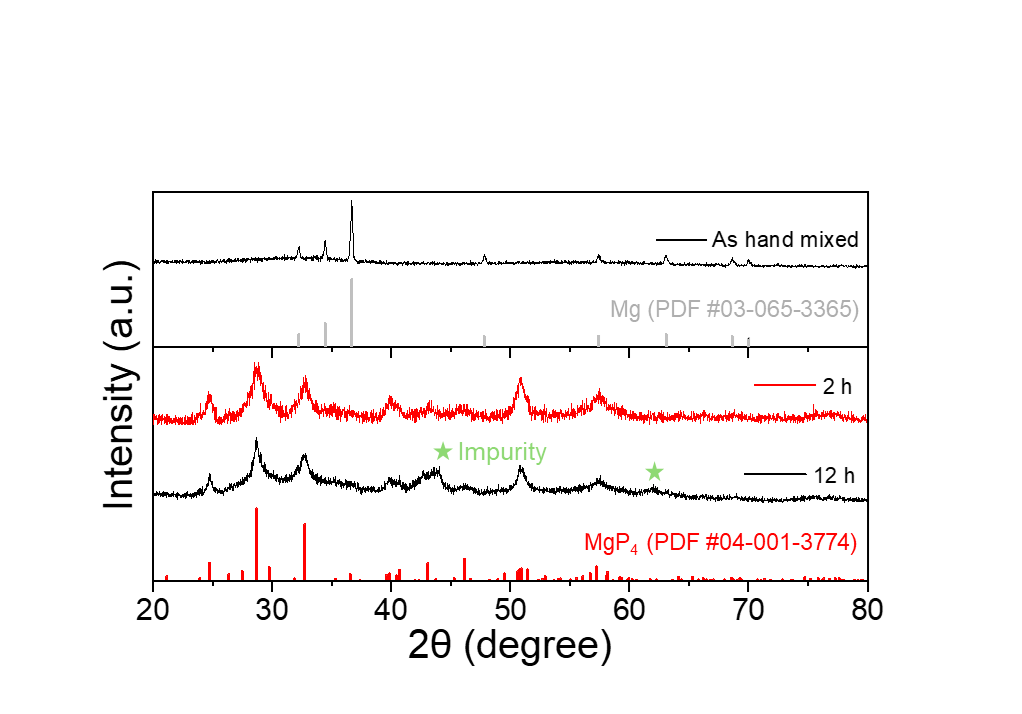


**Figure S1.** X-ray diffraction patterns of MgP_4_ powders prepared by hand mixing and milling for 2 h and 12 h.

**Table S1.** Le Bail fitting result of as-synthesized MgP_4_ particles compared to PDF #04-003-4161 (monoclinic, P2_1_/c).

|  | MgP_4_ | MgP_4_ (PDF #04-003-4161) |
| --- | --- | --- |
| Crystal system | Monoclinic | Monoclinic |
| Space group | P2_1_/c | P2_1_/c |
| a (Å) | 5.1267(5) | 5.1415(3) |
| b (Å) | 5.1037(9) | 5.0791(10) |
| c (Å) | 7.5256(9) | 7.5177(5) |
| α (°) | 90 | 90 |
| β (°) | 98.667(5) | 98.644(4) |
| γ (°) | 90 | 90 |
| R_wp_ (%) | 6.07 | - |


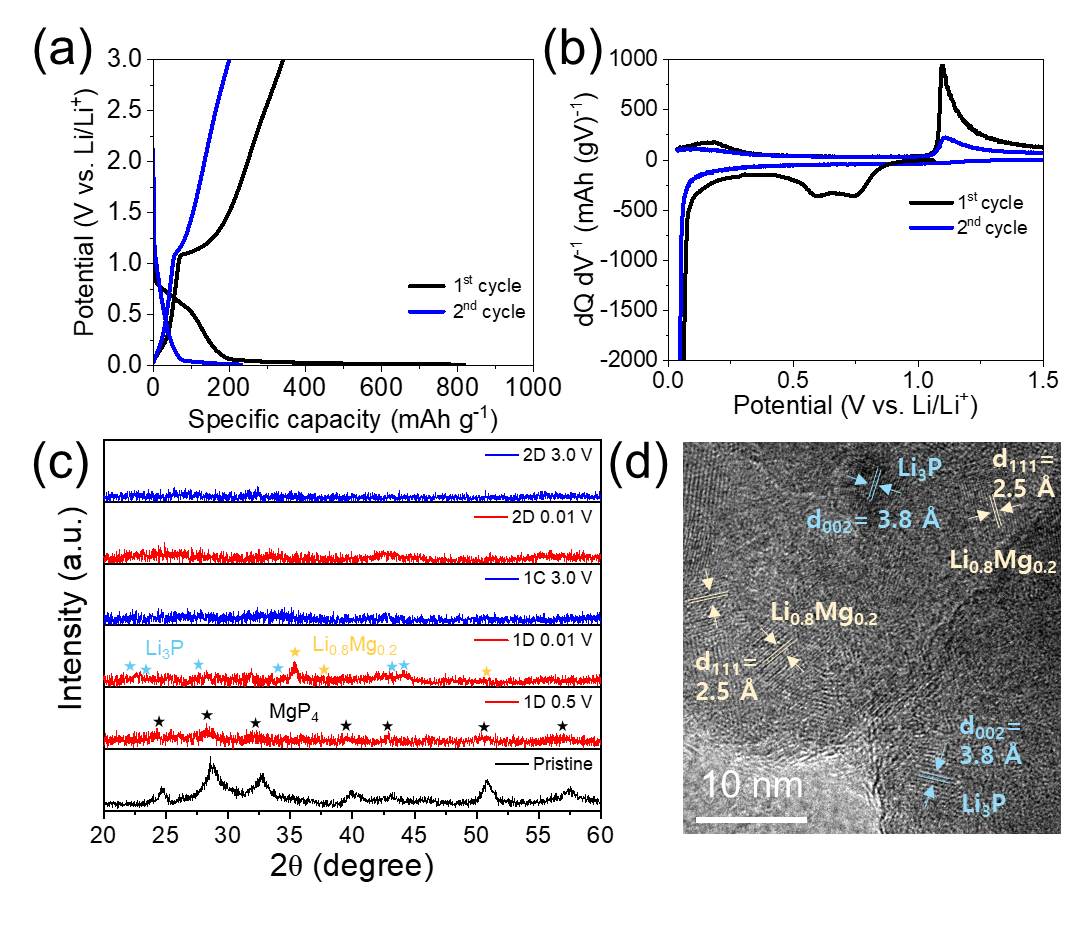
**Figure S2.** (a) Galvanostatic discharge/charge voltage profiles, (b) corresponding differential capacity plots (DCPs), and (c) ex situ XRD patterns at different discharge and charge states of MgP_4_ electrodes for LIBs. (d) HRTEM image for the 1^st^ discharged state of MgP_4_ electrode. The reference peaks corresponding to MgP_4_ (PDF #04-003-4161), Li_3_P (PDF #00-221-2780), and Li_0.8_Mg_0.2_ (PDF #00-152-3818) phases are marked with black, sky-blue, and yellow stars, respectively.


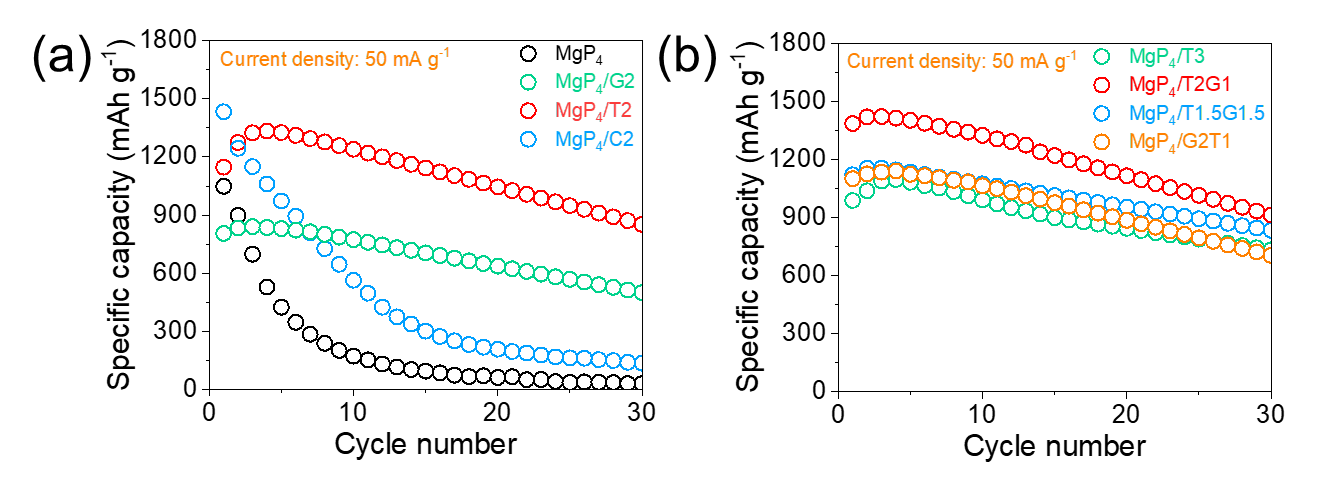


**Figure S3.** Cycle performance of (a) pristine MgP_4_ and MgP_4_ composite electrodes containing 20 wt.% carbon (MgP_4_/G2, MgP_4_/T2, and MgP_4_/C2), and (b) MgP_4_ composite electrodes containing 30 wt.% carbon (MgP_4_/T3, MgP_4_/T2G1, MgP_4_/T1.5G1.5, and MgP_4_/G2T1) at a current density of 50 mA g^−1^ for SIBs. The used electrolyte was 1.0 M NaClO_4_ in EC/DMC (1:1, v/v) with 5 vol.% FEC.


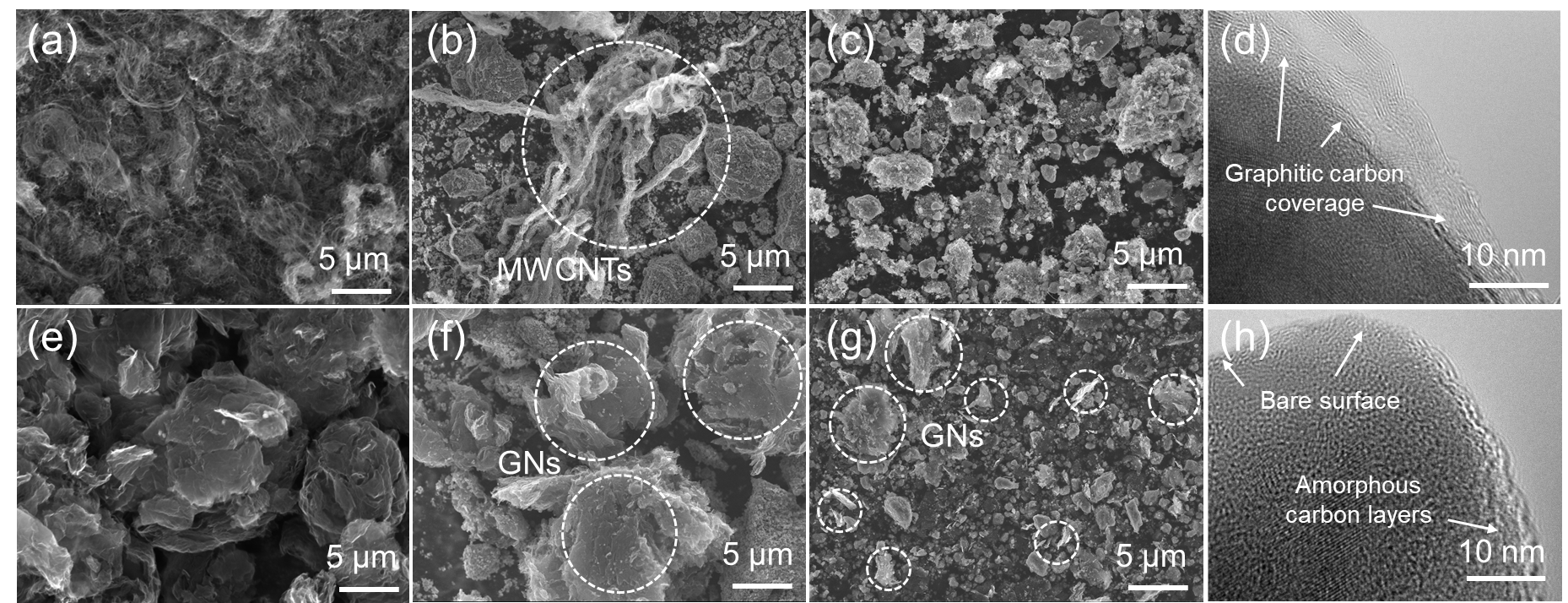


**Figure S4.** SEM images of (a) Pristine MWCNTs, (b) hand-mixed MgP_4_/T2, (c) ball-milled MgP_4_/T2, and (d) TEM image of (c). (e) Pristine GNs, (f) hand-mixed MgP_4_/G2, (g) ball-milled MgP_4_/G2, and (h) TEM image of (g), respectively.


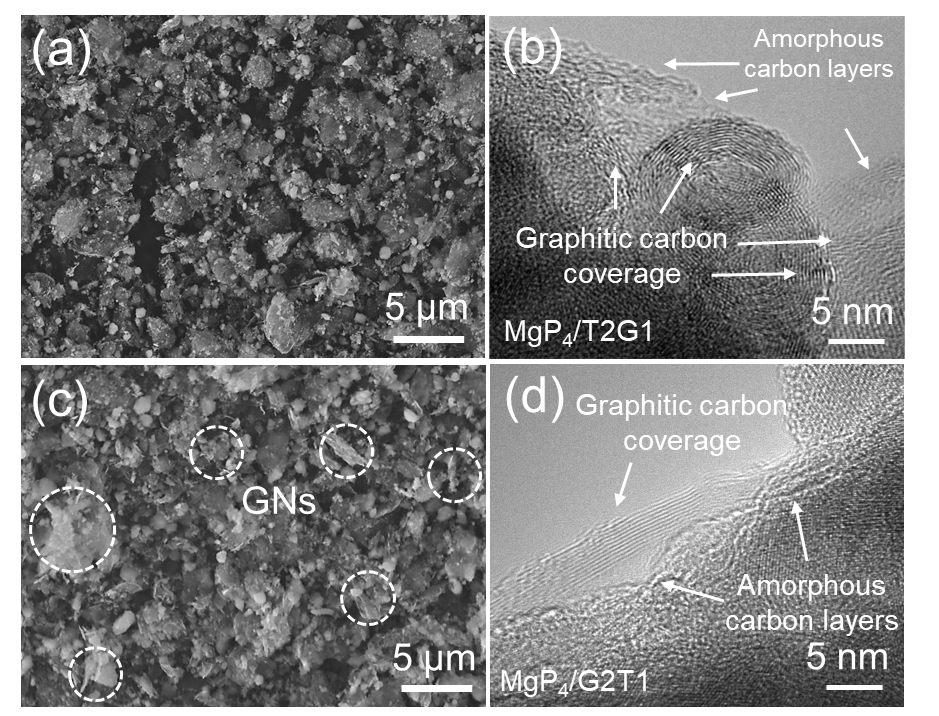


**Figure S5.** SEM and TEM images of (a,b) MgP_4_/T2G1 and (c,d) MgP_4_/G2T1, respectively.

**Table S2.** Comparison of the electrochemical performance with state-of-the-art phosphide anodes for SIBs.

| Material | Mass loading (mg cm^-2^) | Electrolyte | Voltage window (V vs. Na/Na^+^) | Reversible capacity (mAh g^−1^) | Current density (mA g^−1^) | Cycle number (N) | Ref. |
| --- | --- | --- | --- | --- | --- | --- | --- |
| CuP_2_/C | 2.0 | 1 M NaClO_4_ in EC/PC  (1:1, v/v) with 5 vol.% FEC | 0.01−2.0 V | 477 | 0.1 | 200 | [1] |
| FeP@C/rGO | 2.0−3.0 | 1 M NaPF_6_ in EC/DEC  (1 : 1, v : v) with 2% FEC | 0.01−2.5 V | 450.5 | 0.2 | 1000 | [2] |
| CrP_4_/C | ~1.0 | 1 M NaClO_4_ in EC/DMC  (1 : 1, v/v) with 5 vol.% FEC | 0.01−2.5 V | 369 | 0.5 | 100 | [3] |
| GeP_5_/AB/p-rGO | 1.5 | 1 M NaClO_4_ in EC/PC  (1:1, v/v) with 5% FEC | 0.01−2.8 | 400 | 0.5 | 50 | [4] |
| FeP_4_@C-40 | 1.3 | 1 M NaClO_4_ in PC  with 5 wt.% FEC | 0.01−3 V | 205 | 0.5 | 1000 | [5] |
| Ni–CoP@C–N⊂CF | - | 1 M NaClO_4_ in PC  with 5 vol.% FEC | 0.01−3.0 V | 299.7 | 0.5 | 1000 | [6] |
| MnP_4_/G | 1.8−2.2 | 1 M NaClO_4_ in EC/DMC  (1:1, v/v) with 5 vol.% FEC | 0.01−2.0 V | 446 | 0.5 | 250 | [7] |
| ZnP_2_-C | ~2.5 | 0.7 M NaClO_4_ in EC/DEC(1:1, v/v) with 5% FEC | 0−2.0 V | 500 | 0.9 | 200 | [8] |
| FL-GeP/rGO | 1.0 | 1 M NaClO_4_ in EC/DEC  (1:1, v/v) with 5% FEC | 0.01−2.5 V | 230 | 1.0 | 250 | [9] |
| Sn_4_P_3_@CNF | - | 1 M NaPF_6_ in EC/DMC  with 10%FEC | 0.01−3.0 V | 336 | 1.0 | 500 | [10] |
| NiP_3_/CNT | 0.4−0.9 | 1 M NaClO_4_ in EC/DEC  (1:1, v/v) with 5 wt.% FEC | 0.005−1.5 V | 363.8 | 1.6 | 400 | [11] |
| MgP_4_/T2G1 | 1.0−1.5 | 1 M NaClO_4_ in EC/DMC  (1 : 1, v/v) with 5 vol.% FEC | 0.1−1.5 V | 468.5 | 1.0 | 500 | This  work |

1. J. Duan, S. Deng, W. Wu, X. Li, H. Fu, Y. Huang, W. Luo, *ACS Appl. Mater. Interfaces* **2019**, *11*, 12415–12420.
2. Y. Wang, Y. V. Lim, S. Huang, M. Ding, D. Kong, Y. Pei, T. Xu, Y. Shi, X. Li, H. Y. Yang, *Nanoscale* **2020**, *12*, 4341-4351.
3. J. Lee, D. Lee, K.-H. Kim, S.-H. Hong, *J. Mater. Chem. A* **2024**, *12*, 11463-11472.
4. Q.-L. Ning, B.-H. Hou, Y.-Y. Wang, D.-S. Liu, Z.-Z. Luo, W.-H. Li, Y. Yang, J.-Z. Guo, X.-L. Wu, *ACS Appl. Mater. Interfaces* **2018**, 10, 36902-36909.
5. X. Wang, H. Li, C. Xu, H. Sun, C. Fan, W. Song, H. Li, J. Gao, Z. Liu, Y. He, *J. Alloys Compd.* **2022**, *901*, 163577.
6. H. Li, X. Wang, Z. Zhao, R. Pathak, S. Hao, X. Qiu, Q. Qiao, *J. Mater. Sci. Technol.* **2022**, *99*, 184-192.
7. K.-H. Kim, S.-H. Hong, *Adv. Energy Mater*. **2021**, *11*, 2003609.
8. K.-H. Nam, Y. Hwa, C.-M. Park, *ACS Appl. Mater. Interfaces* **2020***, 12, 15053–15062*.
9. Y. Fuhua, H. Jian, H. Junnan, Z. Shilin, L. Gemeng, L. Jun, L. Yuqing, L. Nana, P. Wei Kong, C. Jun, G. Zaiping, *Adv. Energy Mater*. **2020**, *10*, 1903826.
10. L. Ran, I. Gentle, T. Lin, B. Luo, N. Mo, M. Rana, M. Li, L. Wang, R. Knibbe, *J. Power Sources* **2020**, *461*, 228116.
11. M. I.-U.-Haq, H. Huang, J. Cui, S. Yao, J. Wu, W. G. Chong, B. Huang, J.-K. Kim, J. Mater. Chem. A, 2018, 6, 20184-20194.


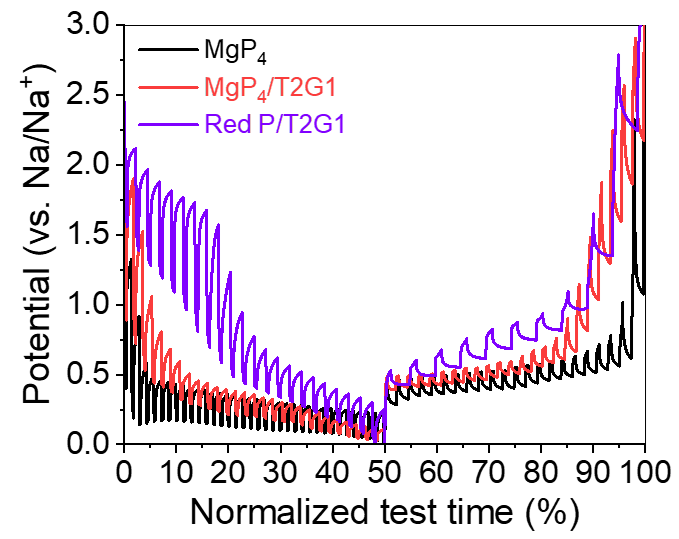


**Figure S6.** GITT curves of MgP_4_, MgP_4_/T2G1, Red P/T2G1 electrodes. The used electrolyte was 1.0 M NaClO_4_ in EC/DMC (1:1, v/v) with 5 vol.% FEC.

**
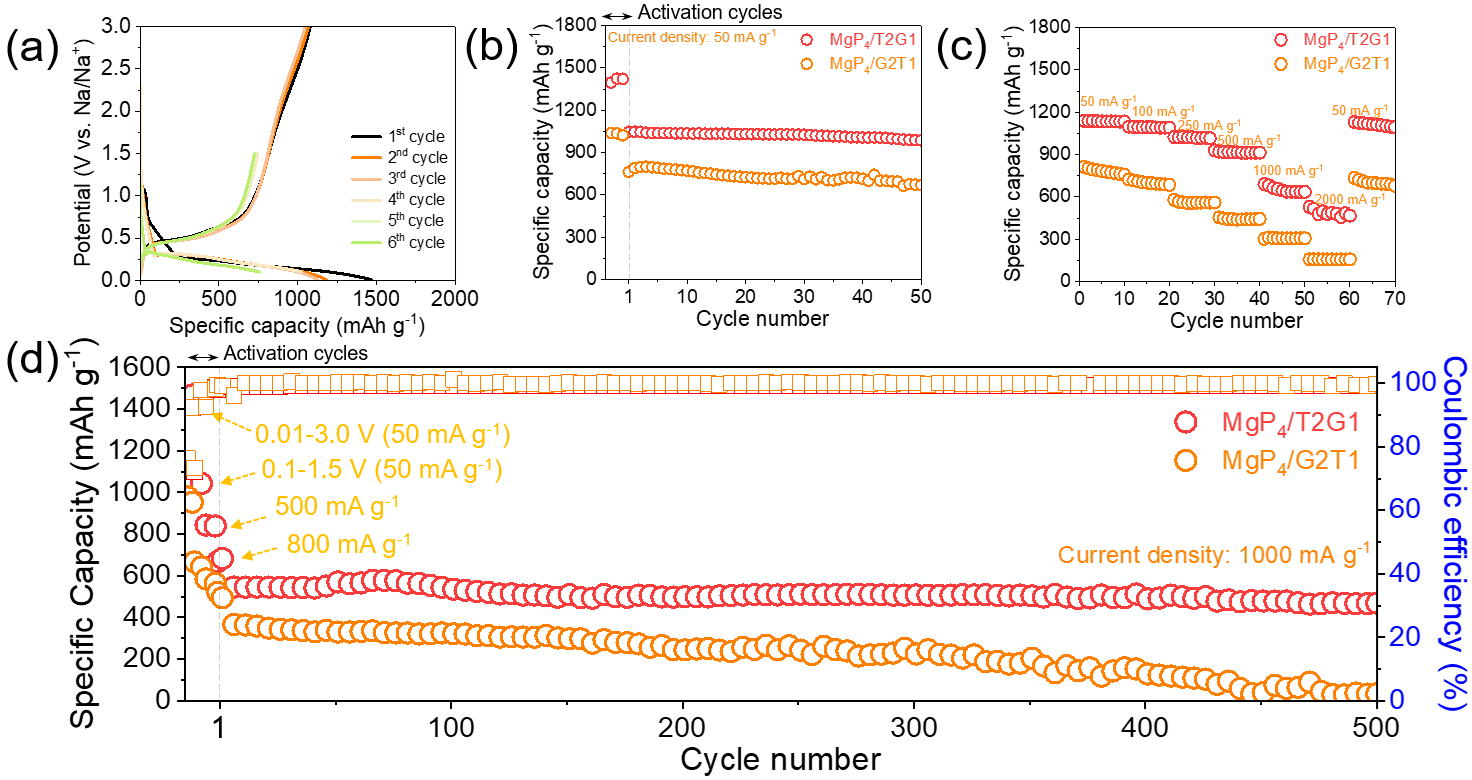
Figure S7.** (a) Galvanostatic discharge/charge voltage profiles of MgP_4_/G2T1 electrode. (b) Cycle performance tested at 50 mA g^−1^, (c) rate capability, and (d) long-term cycle performance tested at 1000 mA g^−1^ for MgP_4_/T2G1 and MgP_4_/G2T1 electrodes in SIBs. The used electrolyte was 1.0 M NaClO_4_ in EC/DMC (1:1, v/v) with 5 vol.% FEC.


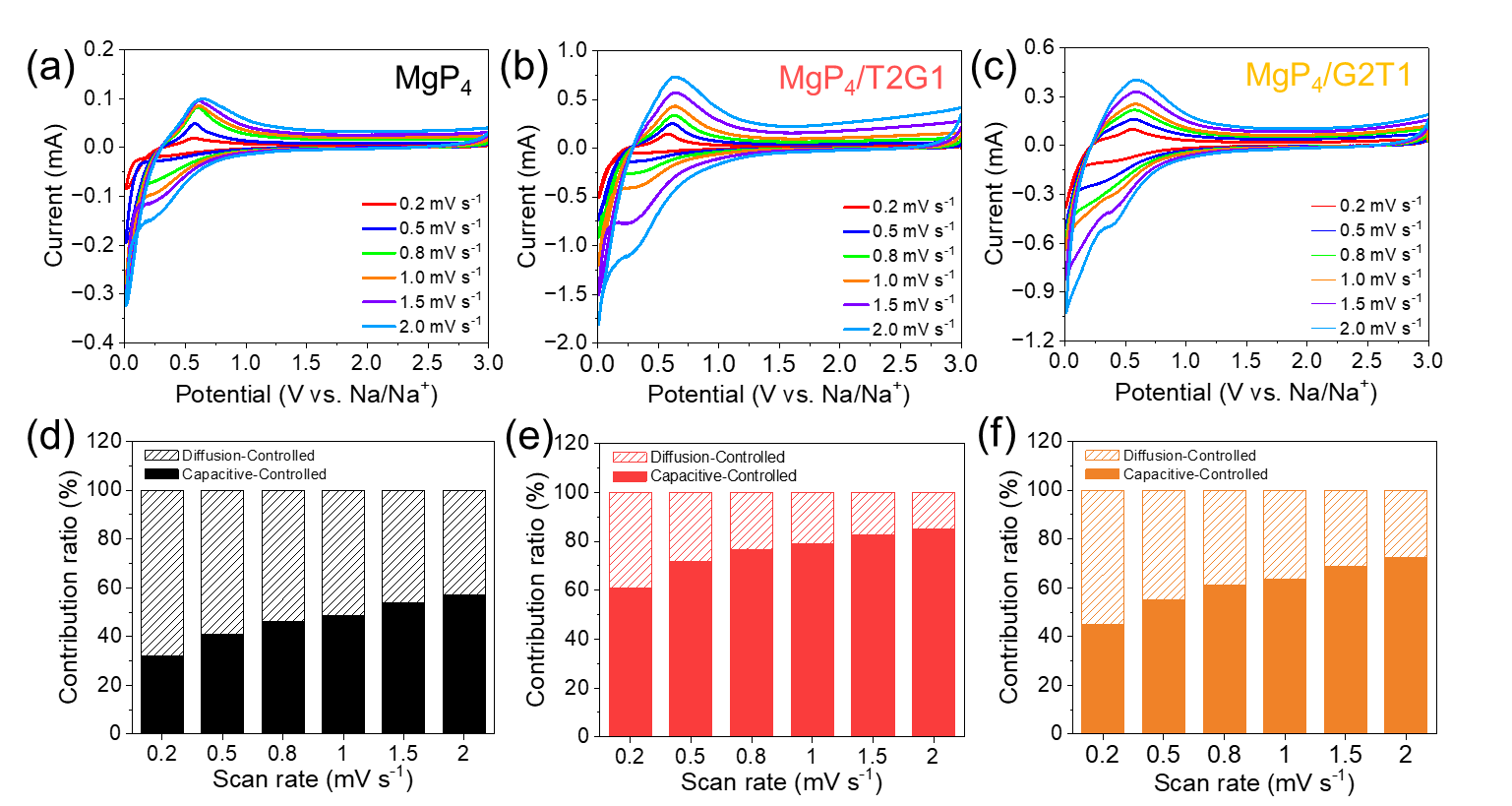
**Figure S8.** Cyclic voltammetry curves at various scan rates from 0.2-2.0 mV s^−1^ and corresponding normalized contribution ratio of (a,d) MgP_4_ electrode, (b,e) MgP_4_/T2G1, and (c,f) MgP_4_/G2T1 electrodes for SIBs, respectively. The used electrolyte was 1.0 M NaClO_4_ in EC/DMC (1:1, v/v) with 5 vol.% FEC.

**
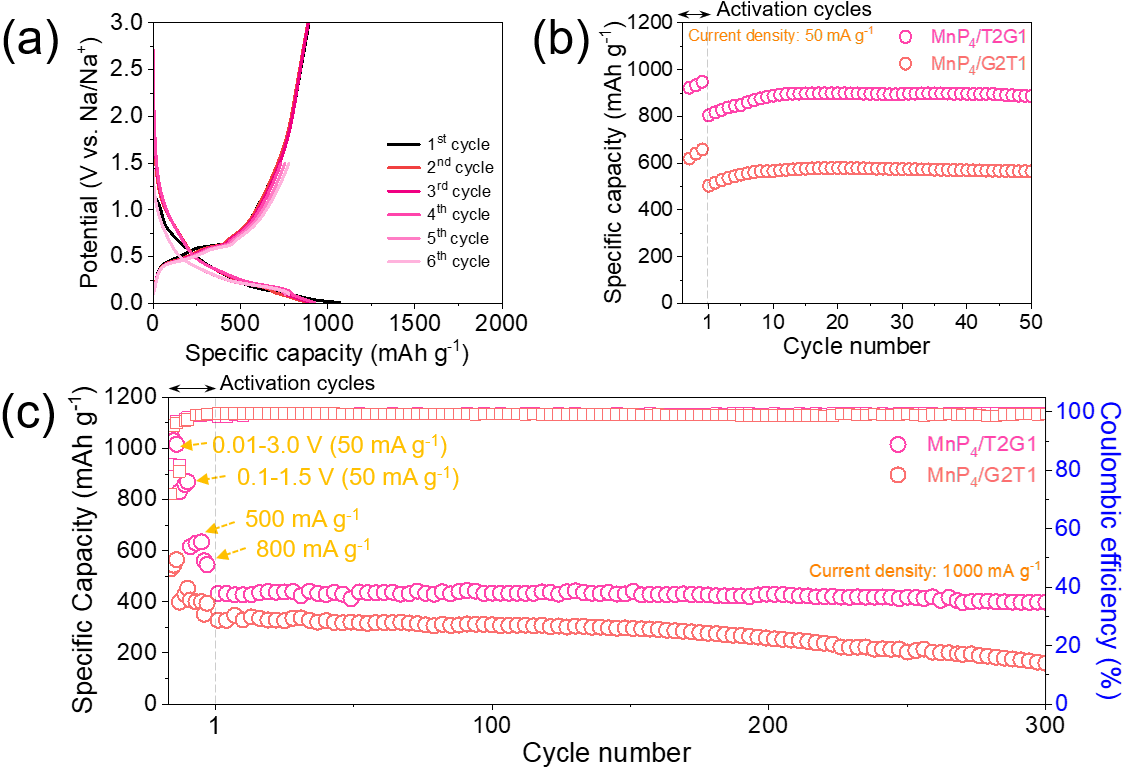
Figure S9.** Galvanostatic discharge/charge voltage profiles of MnP_4_/G2T1 electrode. (b) Cycle performance tested at 50 mA g^−1^ and (c) long-term cycle performance tested at 1000 mA g^−1^ for MnP_4_/T2G1 and MnP_4_/G2T1 electrodes in SIBs. The used electrolyte was 1.0 M NaClO_4_ in EC/DMC (1:1, v/v) with 5 vol.% FEC.


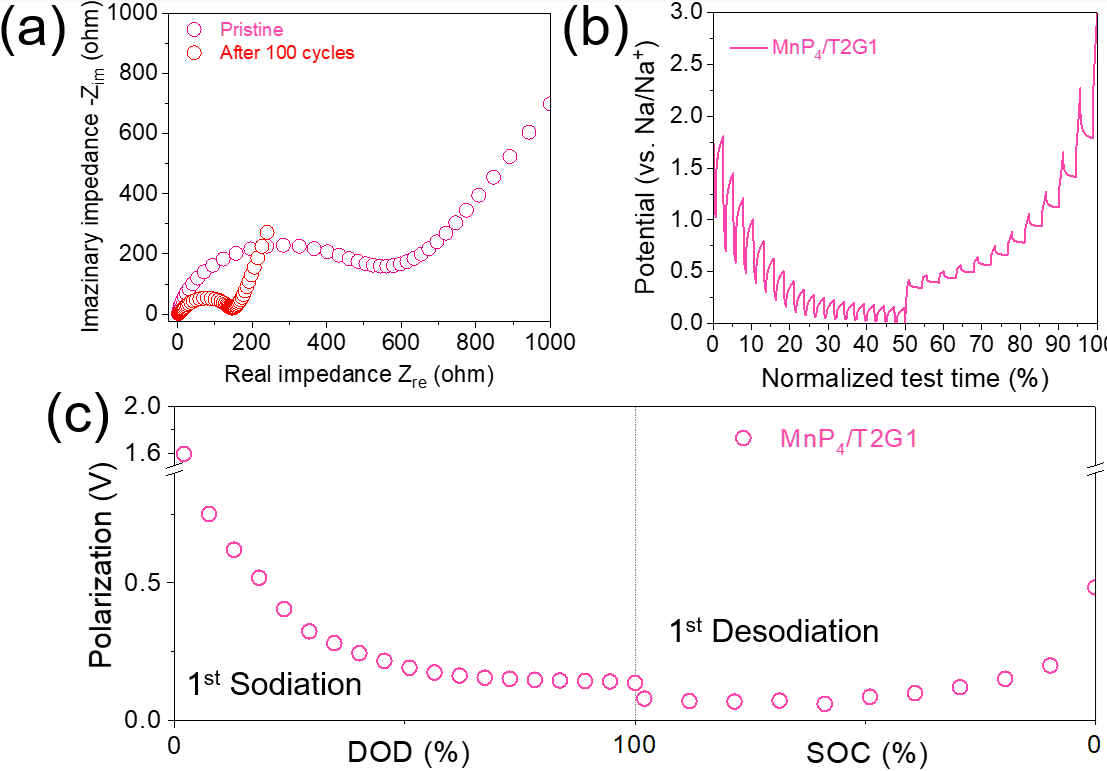
**Figure S10.** (a) Nyquist plots of before and after 100 cycles at a current density of 1000 mA g^−1^, (b) GITT curves, and (c) measured polarization from (b) during 1^st^ discharge and charge processes of MnP_4_/T2G1 electrodes, respectively. The used electrolyte was 1.0 M NaClO_4_ in EC/DMC (1:1, v/v) with 5 vol.% FEC.

**Table S3.** Charge transfer resistances of MgP_4_/T2G1, MnP_4_/T2G1, and Red P/T2G1
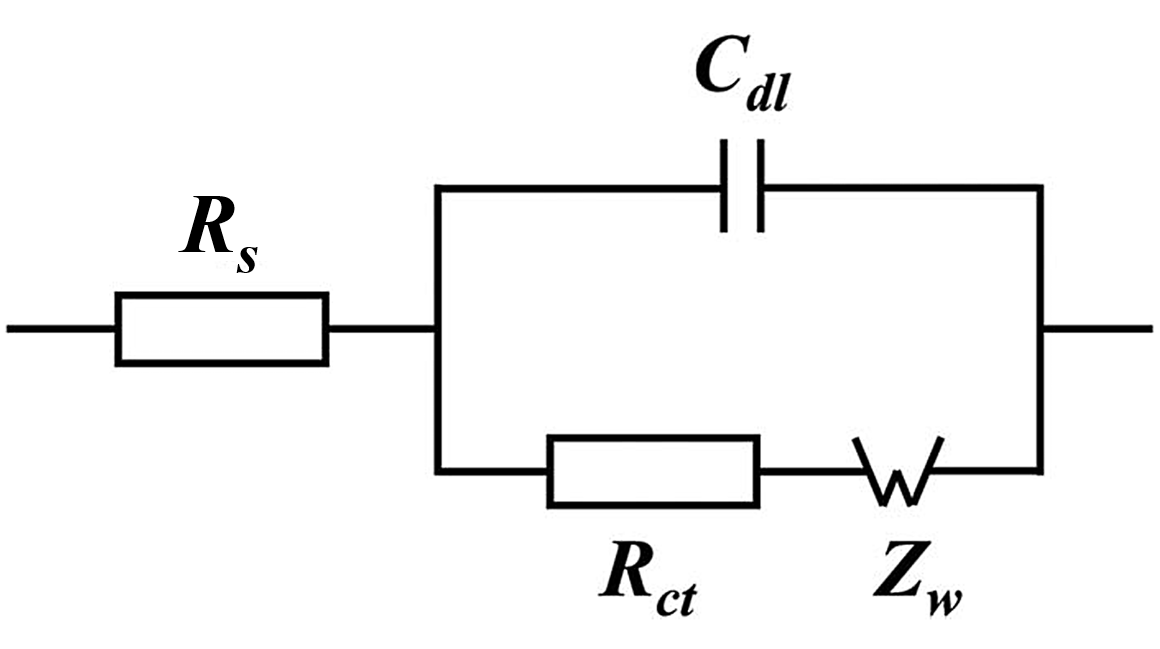
electrodes for before and after 100 cycle test.

| Material | Charge transfer resistance, R_ct_ (Ω) | |
| --- | --- | --- |
|  | Before cycle | After 100 cycles |
| MgP_4_/T2G1 | 350.0 | 105.2 |
| MnP_4_/T2G1 | 602.3 | 149.8 |
| Red P/T2G1 | 440.0 | 238.8 |


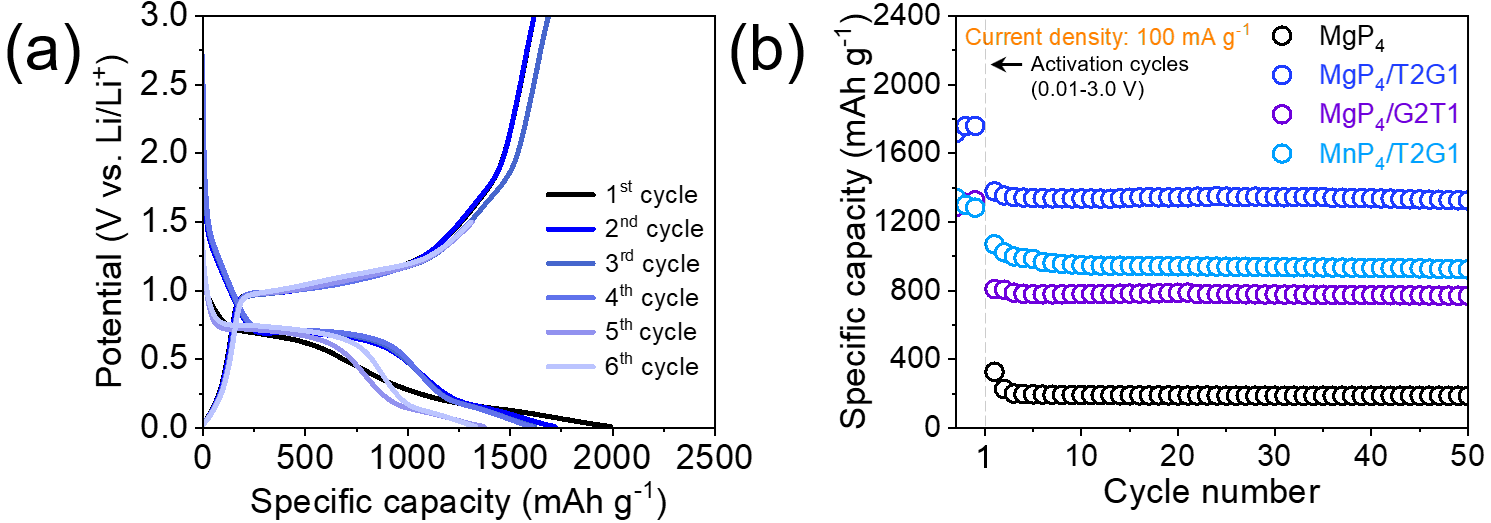


**Figure S11.** (a) Galvanostatic discharge/charge voltage profiles of MgP_4_/T2G1 electrode tested at a current density of 100 mA g^−1^. (b) Cycle performance tested at 100 mA g^−1^ for MgP_4_, MgP_4_/T2G1, MgP_4_/G2T1, and MnP_4_/T2G1 electrodes in LIBs. The used electrolyte was 1.0 M LiPF_6_ in EC/DMC/EMC(1:1:1, v/v) with 10 wt.% FEC.


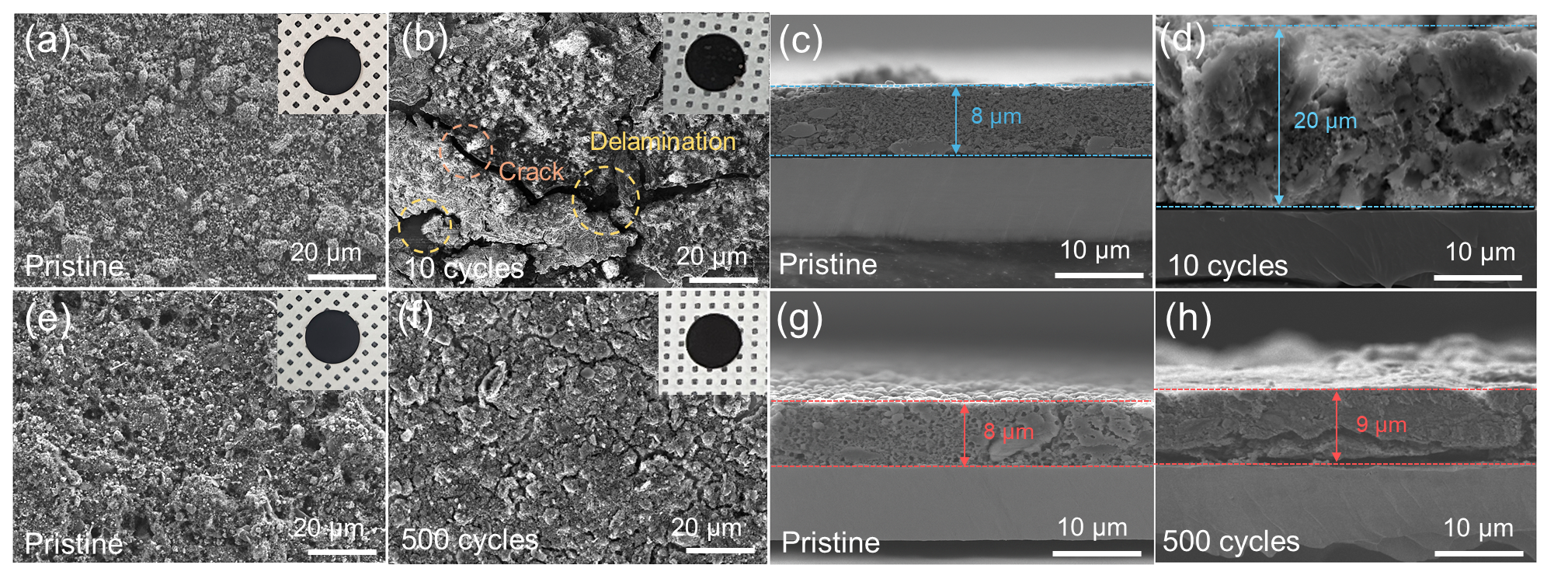


**Figure S12.** Top-view and cross-sectional SEM images of (a-d) MgP_4_ and (e-h) MgP_4_/T2G1 electrodes before and after cycling. The cycled MgP_4_ and MgP_4_/T2G1 electrodes were tested at 1000 mA g^−1^ after 10 and 500 cycles, respectively.


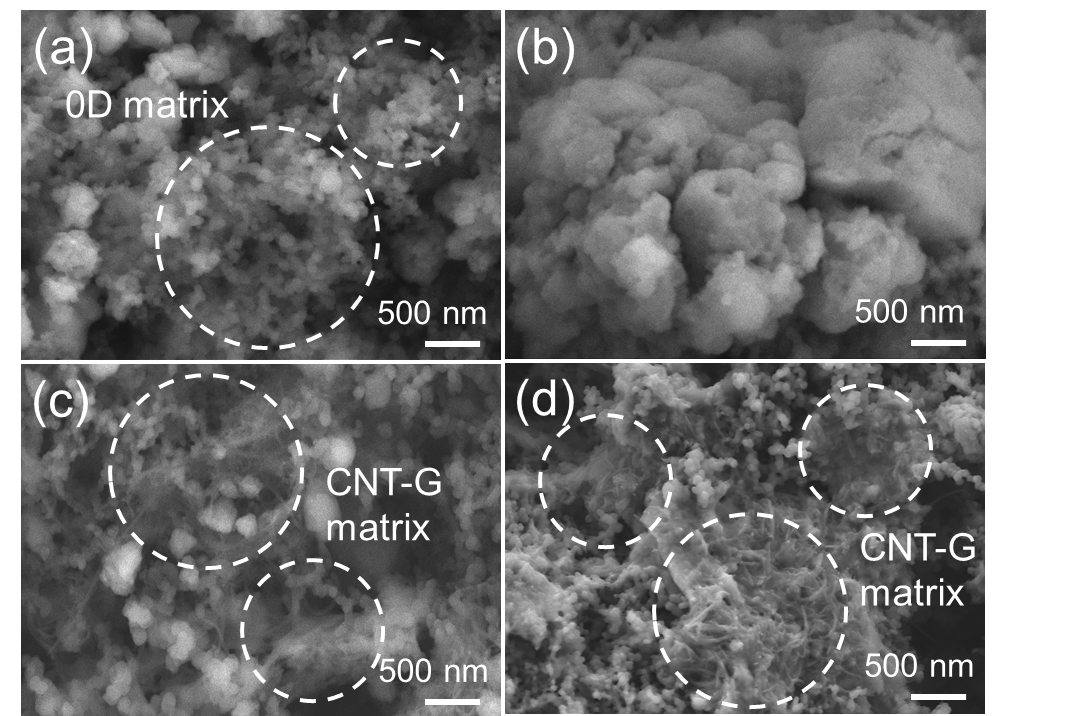


**Figure S13.** SEM images of (a,b) MgP_4_ and (c,d) MgP_4_/T2G1 electrodes before and after cycling. The cycled MgP_4_ and MgP_4_/T2G1 electrodes were tested at 1000 mA g^−1^ after 10 and 500 cycles, respectively.


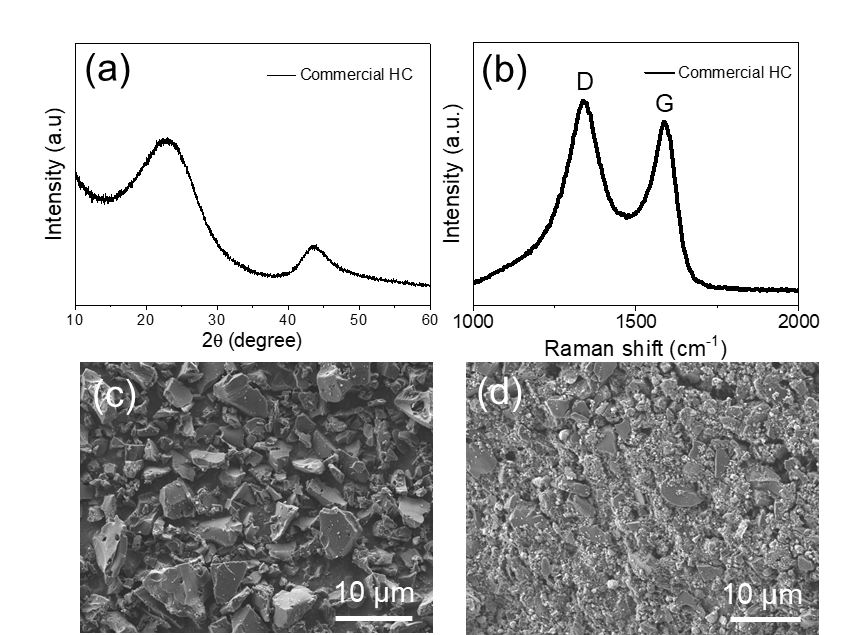


**Figure S14.** (a) XRD pattern, (b) Raman spectrum, and SEM images of (c) commercial hard carbon and (d) MgP_4_/T2G1@HC electrode, respectively.


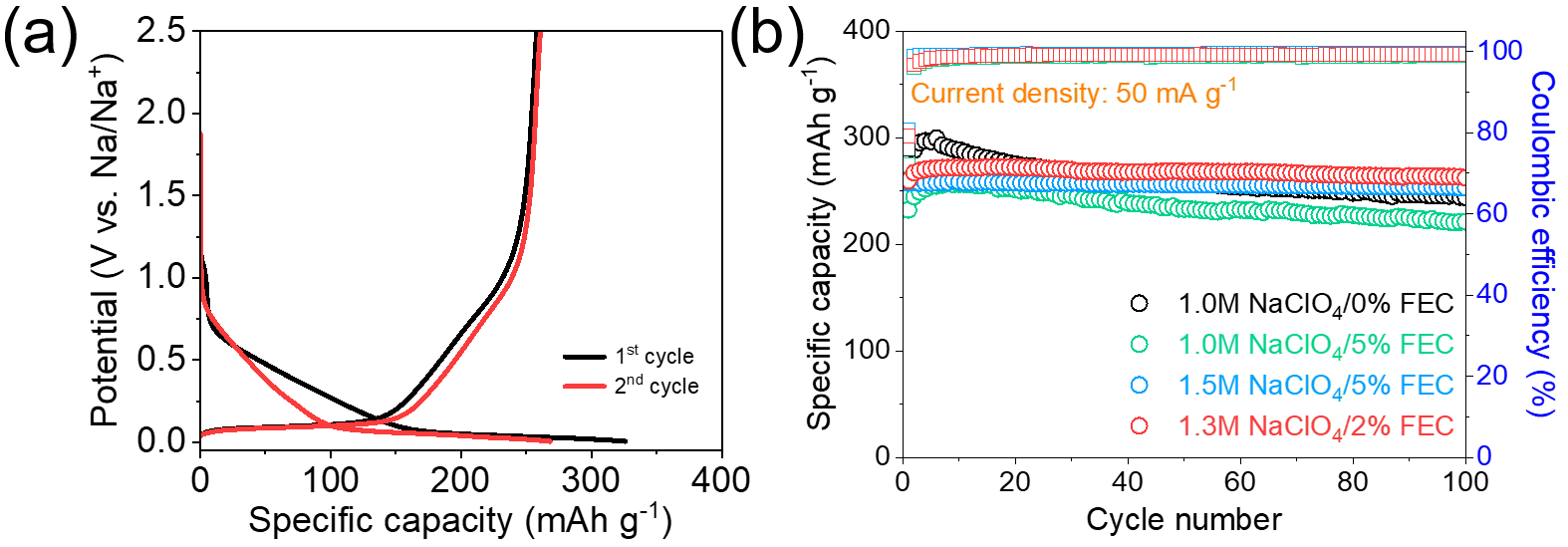
**Figure S15.** (a) Voltage profiles of commercial hard carbon electrodes with the optimized electrolyte (1.3 M NaClO_4_ in EC/DMC(1:1, v/v) with 2 vol.% FEC) and (b) cycle performances with various electrolytes.

**Table S4.** Comparison of the electrochemical performance with state-of-the-art hard carbon anodes for SIBs.

| Material | Electrolyte | Current density (mA g^−1^) | Reversible capacity (mAh g^−1^) | Cycle number  (N) | Ref. |
| --- | --- | --- | --- | --- | --- |
| MgP_4_/T2G1@HC | 1.3 M NaClO_4_ in EC:DMC (1:1, v/v) with 2 vol.% FEC | 1000 | 146.5 | 2000 | This work |
| PPHC900 | 1.0 M in NaClO_4_ in EC:DMC (1:1, v/v) | 350 | 120 | 1000 | [12] |
| HCH-600 | 1.0 M NaClO_4_ in EC:DEC (1:1, v/v) | 300 | 150 | 300 | [13] |
| HHC | 1.0 M NaClO_4_ in EC:PC (1:1, v/v) | 500 | 80 | 600 | [14] |
| L6-1300 | 1.0 M NaPF_6_ in EC:DEC (1:1, v/v) | 300 | 237 | 500 | [15] |
| CMAC-2 | 1.0 M NaClO_4_ in EC:DEC (1:1, v/v) | 300 | 79.6 | 500 | [16] |
| PCLC-1 | 1.0 M NaClO_4_ in EC:DEC (1:1, v/v) with 5% FEC | 300 | 246.8 | 1000 | [17] |
| PRC-81-1300 | 1.0 M NaPF_6_ in EC:DMC | 1000 | 98.0 | 1000 | [18] |
| VHC-1200 | 1.0 M NaPF_6_ in diglyme | 500 | 200 | 1000 | [19] |
| CC-1400 | 1.0 M NaPF_6_ in DME | 1000 | 190 | 300 | [20] |
| TS-1400-HCl | 1.0 M NaCF_3_SO_3_ in diglyme | 280 | 200 | 500 | [21] |
| Balling-12h-1500 | 1.0 M NaPF_6_ in DME | 500 | 230 | 500 | [22] |
| MFCP-1300 | 1.0 M NaPF_6_ in diglyme | 1000 | 220 | 500 | [23] |

- - 1. K. Bhawana, M. Gautam, G. K. Mishra, N. Chakrabarty, S. Wajhal, D. Kumar, D. P. Dutta, S. Mitra, *Carbon.* **2023**, *214*, 118319.
    2. X. Lou, Y. Cao, S. Guo, H. Han, X. Jiang, S. Siqing, Z. long, X. Zhu, X. Qiu, *J. Electrochem. Soc.* **2023**, *170*, 120510.
    3. H. Xie, Z. Wu, Z. Wang, N. Qin, Y. Li, Y. Cao, Z. Lu, *J. Mater. Chem. A*, **2020***, 8*, 3606-3612.
    4. Z. Tang, D. Jiang, Z. Fu, J. Zhou, R. Liu, R. Zhang, D. Sun, A. S. Dhmees, Y. Tang, H. Wang, *Small Methods* **2024**, *8*, 2400509.
    5. S. Zhang, N. Sun, X. Li, R. A. Soomro, B. Xu, *Energy Storage Mater.* **2024**, *66*, 103183.
    6. H. Chen, N. Sun, Y. Wang, R. A. Soomro, B. Xu, *Energy Storage Mater.* **2023**, *56*, 532–541.
    7. Q. Wang, G. Li, R. Li, H. Zhao, C. Chen, L. Wang, Y. Yu, Y. Pan, C. Wang, *J. Power Sources* 2025, *648*, 237423.
    8. G. Pan, R. Zhao, Z. Huang, C. Cui, F. Wang, Y. Gu, Y. Gao, Z. Sun, T. Zhang, *Carbon* **2024,** *224*, 118955.
    9. R. Ma, Y. Chen, Q. Li, B. Zhang, F. Chen, C. Leng, D. Jia, N. Guo, L. Wang, *Chem. Eng. J* **2024,** *493*, 152389.
    10. Q. He, H. Chen, X. Chen, J. Zheng, L. Que, F. Yu, J. Zhao, Y. Xie, M. Huang, C. Lu, J. Meng, X. Zhang, *Adv. Funct. Mater.* **2024**, *34*, 2310226.
    11. Z. Tang, R. Liu, D. Jiang, S. Cai, H. Li, D. Sun**,** Y. Tang, H. Wang, *ACS Appl. Mater. Interfaces* **2024**, *16*, 47504–47512.
    12. Q. Ren, J. Wang, L. Yan, W. Lv, F. Zhang, L. Zhang, B. Liu, Z. Shi, *Chem. Eng. J* **2021**, *425*, 131656.


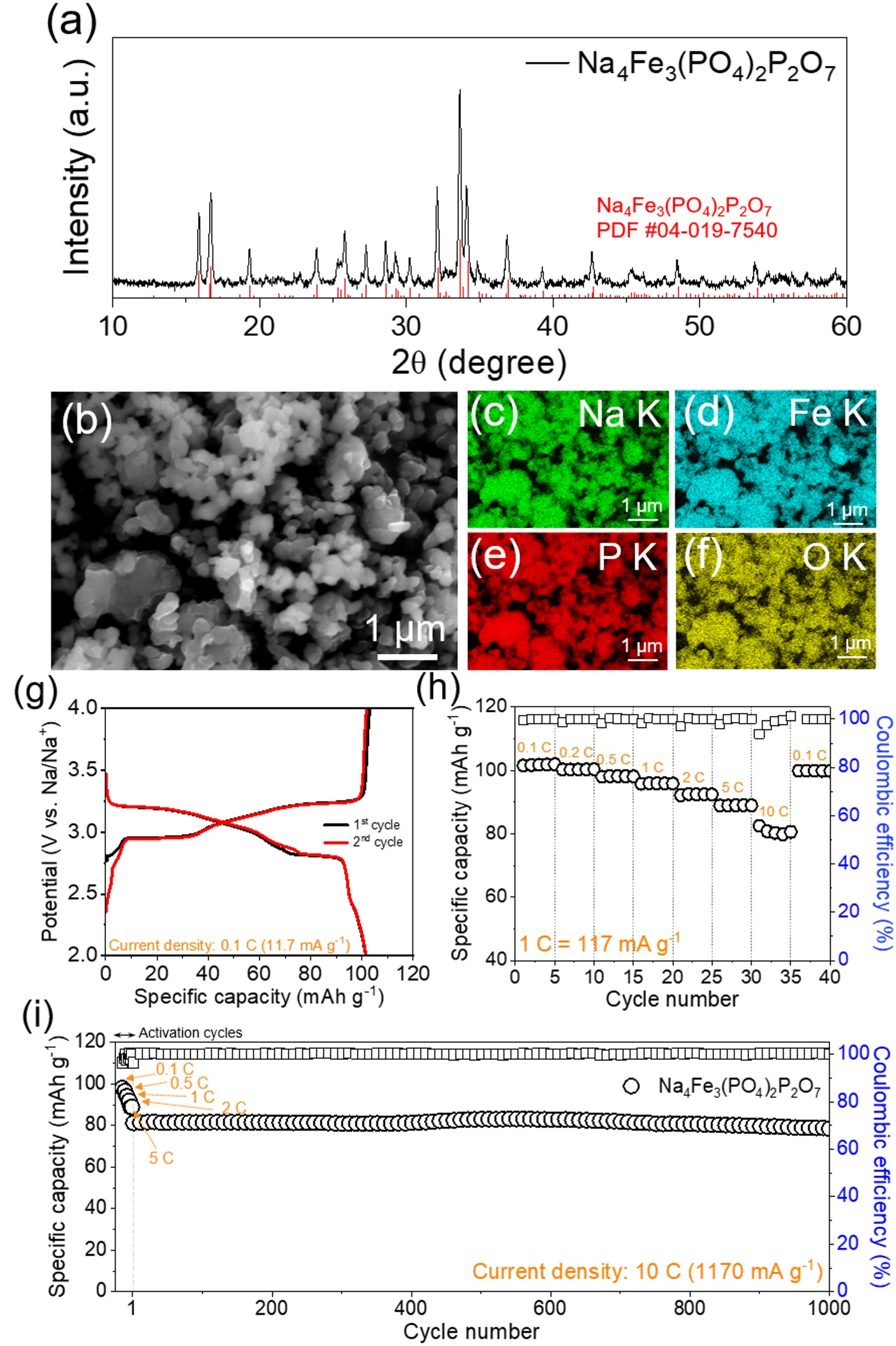


**Figure S16.** (a) XRD pattern, (b) SEM image, corresponding EDS mapping images of (c) Na K, (d) Fe K, (e) P K, and (f) O K of commercial Na_4_Fe_3_(PO_4_)_2_P_2_O_7_ powder. (g) Galvanostatic charge/discharge voltage profiles, (h) rate capability, and (i) long-term cycle performance tested at 10 C of Na_4_Fe_3_(PO_4_)_2_P_2_O_7_ cathode electrode. The used electrolyte was 1.3 M NaClO_4_ in EC/DMC (1:1, v/v) with 2 vol.% FEC.
